# Sustainable service quality assessment of Chinese healthcare e-government: a multi-criteria decision framework based on SERVQUAL model and entropy-weight TOPSIS method

**DOI:** 10.3389/fdgth.2025.1611979

**Published:** 2025-09-08

**Authors:** Yilin Xia, Bei Li, Jian Yang, Weixi Guo, Jianwei Sun, Xu Zeng, Jing Zheng

**Affiliations:** ^1^ Department of Biomedical Informatics, School of Life Science, Central South University, Changsha, China; ^2^ Shenzhen Health Development Research and Data Management Center, Shenzhen, China

**Keywords:** healthcare e-government, SERVQUAL, TOPSIS, MCDM, SDGs

## Abstract

**Introduction:**

Healthcare e-government plays a vital role in advancing the United Nations 2030 Agenda for Sustainable Development, by disseminating vital information and delivering essential services, particularly during the coronavirus disease 2019 (COVID-19) pandemic. China faces challenges within the context of health care, such as limited infrastructure and equitable healthcare access, underscoring the necessity of evaluating and improving the quality of healthcare e-government services.

**Methods:**

This study proposes a novel multicriteria decision-making framework for systematically evaluating the quality of healthcare e-government services. The framework structurally extends the SERVQUAL model into 5 first-level and 31 s-level dimensions tailored to digital governance and integrates it with the entropy weight method and the technique for order of preference by similarity to ideal solution. This hybrid methodology enables objective, scalable, and theory-based assessment. To validate its effectiveness, the model was empirically applied to 17 Chinese municipal health commission websites in the period of 2019–2024 to validate its effectiveness.

**Results:**

The results revealed that empathy represents the most critical quality gap among the evaluated healthcare e-government services, while cross-platform interoperability has become a strategic focus for system improvement. These results highlighted that sustainable service quality is contingent on both robust technical infrastructure and human-centric factors.

**Discussion:**

The proposed framework can offer policymakers a replicable decision-support tool by bridging the gap between operational performance and sustainable service delivery, particularly in developing regions. This provides practical insights into achieving digital healthcare transformation, contributing to the United Nations Sustainable Development Goals, offers a scalable evaluation approach for comparative research in digital governance, and promotes global e-government systems.

## 1 Introduction

Healthcare e-government initiatives play an increasingly vital role in accelerating the achievement of the United Nations 2030 Agenda for Sustainable Development (1). They have supported governments worldwide during healthcare crises, ensuring the effective delivery of essential public services during periods marked by increased isolation, uncertainty, and societal vulnerability. They have become a pivotal tool for communication and collaboration between policymakers and society, a role that was particularly evident during the coronavirus disease 2019 (COVID-19) pandemic when governments worldwide relied on healthcare e-government systems to disseminate critical information, provide services, and develop tools for tracking the pandemic's progress and coordinating relief efforts. Approximately 90% of Member States have established dedicated portals or created specialized sections within their national e-government platforms to address pandemic-related issues and provide relevant public services (2). The 2024 United Nations E-Government Survey paints a picture of progress in the global development of healthcare e-government, with all regions adopting digital technologies to enhance service delivery and public engagement. This trend has accelerated during the post-pandemic world, marked by increased investments in resilient infrastructure and advanced solutions such as cloud computing and broadband (3).

In 2024, the E-Government Development Index (EGDI) of China was 0.8718, ranking 35th globally. This places China in the high-level V3 category. This significant improvement is attributed to strategic policies, substantial investment in digital infrastructure, and innovative initiatives. However, China still lags behind several Asian countries with very high EGDI, namely, Singapore (0.969), South Korea (0.9679), Saudi Arabia (0.9602), the United Arab Emirates (0.9533), and Japan (0.9351). This is due to the fact that local governments are restricted by inadequate internet coverage and insufficient infrastructure that hinder them from delivering efficient and accessible e-government services, thereby affecting both service quality and fairness (3). Furthermore, local healthcare e-government platforms exhibit persistent challenges in reaching vulnerable populations (4), enhancing user experience (5), and improving technological integration (6). Like other segments of the public sector, government health departments, national agencies responsible for monitoring and improving public health, and publicly funded healthcare providers face growing pressure to enhance efficiency, transparency, and service delivery, necessitating their active participation in the e-government initiatives.

While there has been significant progress in e-government in the healthcare sector, systematic evaluations of service quality within this domain remain relatively insufficient. Extant studies primarily focus on macro-level infrastructure, policy implementation, and information disclosure (7–9) but do not offer fine-grained, multidimensional assessments of local health department e-government platforms. The few studies that have evaluated service quality typically rely on a limited number of dimension indices or expert-assigned weights (10, 11), lacking an integrated user-centered experience aspect such as accessibility, interactivity, interface design, and overall usability. This limits data-driven policy improvement and resource optimization. Moreover, although some classic evaluation models have been widely validated in healthcare service assessment (12), their application in the context of e-government and integration with multicriteria decision-making (MCDM) methods remain limited. MCDM techniques, such as technique for order of preference by similarity to ideal solution (TOPSIS) and fuzzy/analytic hierarchy process (AHP)-TOPSIS, have been adopted in public sector evaluations, including the provision of government services and organizational resource planning (13), but are rarely represented in the healthcare e-government domain (14–16). Other countries have some comprehensive SERVQUAL + AHP-TOPSIS frameworks for public services (17, 18), but similar frameworks tailored for local healthcare e-platforms are virtually nonexistent.

Therefore, it is necessary to establish a comprehensive and universal evaluation framework that can integrate the multidimensional characteristics of service quality and has strong data compatibility and interpretability to more scientifically and operatively identify the key improvement priorities in local government healthcare e-government platforms and enhance their service quality.

Herein, an e-government service quality assessment system is developed based on an MCDM framework. The system integrates and expands the original structure of the SERVQUAL model, further refining its five core dimensions into 5 first-level dimensions and 31 s-level dimensions, particularly tailored to the services of the Official Websites of Municipal Health Commissions in China. Due to its theoretical completeness and cross-domain applicability, the SERVQUAL model has been a widely applied tool for measuring service quality in various contexts such as health care, education, and government affairs. Its dimensionalized analysis feature effectively overcomes the fragmented limitations of traditional single-dimensional assessment methods. To enhance the objectivity and data-driven nature of the evaluation system, this study employs the entropy weight method (EW) to allocate weights to each indicator, identifying high-differentiation features based on the principle of information entropy, thereby avoiding bias from subjective weighting. Given that the data collected for this study is sourced from annual government website work reports, which encompasses quantitative information, such as total information releases, and qualitative information, such as page design, this study further introduces the TOPSIS method for data normalization and comprehensive ranking, which is well known in multiattribute decision analysis due to its simplicity and robustness, effectively supporting tasks involving heterogeneous indicators and priority identification. Finally, a service quality ranking map is generated to present the improvement priorities of each website in terms of service quality dimensions.

The contribution of this study lies in proposing and validating a practical, theory-based, and reality-adaptive framework for evaluating the quality of e-government services. Moreover, through empirical research on Chinese cases, its practical value has been verified. It has been proven that this framework has strong applicability and practical value in optimizing the efficiency of digital governance in countries or regions with limited technological infrastructure, fragmented policies, and restricted financial or administrative capabilities. In addition, the framework can be replicated for digital governance assessment under the MCDM paradigm.

The rest of this study is structured as follows. Section 2 presents the related literature review on service quality measurements of healthcare e-government. Section 3 explains the construction process of the research framework. Section 4 introduces a case study. Section 5 extensively discusses the research findings and puts forward policy suggestions and practical implications. Section 6 delineates the conclusions, and Section 7 points out the limitations of this study.

## 2 Literature review

By screening the recent studies on quality assessment of e-government services and utilization of the SERVQUAL model, there exists considerable research on the assessment and development of e-government services; however, studies specifically addressing healthcare e-government services remain limited. Compared with other sectors, implementing e-government initiatives in healthcare faces substantial technological, managerial, and implementation challenges (4). Mainda (19) examined the role of e-government in enhancing service efficiency in healthcare, highlighting that the adoption of digital technologies improves the efficiency, transparency, and accessibility of medical services. They identified system integration, user acceptance, and stakeholder collaboration as critical factors for the success of these initiatives. Anthopoulos et al. (20), in their analysis of the HealthCare.gov website's failure, identified poor project management, technical barriers, changing requirements, and bureaucracy as contributing factors. They emphasized quality assurance, effective planning, and stakeholder engagement for successful projects. Sorn-in et al. (21) examined key factors influencing e-government development and revealed that service quality, policy-and-governance frameworks, information technology infrastructure, organizational factors, and socioeconomic conditions play crucial roles. Governments should prioritize them to meet citizen demands and continuously improve e-government services. Wang et al. (22) stressed that bidirectional, instantaneous information exchange is vital for optimizing healthcare governance: the public accesses authoritative health guidelines (e.g., vaccination and pandemic prevention measures) via government platforms, thereby reducing health risks, while governments gather real-time public health data (e.g., self-reported symptoms and regional infection rates) through official platforms and social media channels to inform evidence-based decision-making. Recent empirical work from Tanzania further illustrates that perceived usefulness and ease of use are decisive for medical staff acceptance of digital platforms (19), while provincial panel data from China confirm that integrated e-government significantly improves both the quality and the equity of public services through efficiency and innovation channels (23).

SERVQUAL is a widely recognized model for service evaluation in five dimensions, namely, tangibility, reliability, responsiveness, assurance, and empathy (24). This model is particularly effective in assessing service fairness, coverage, and technical compatibility (25). Moreover, it can identify specific service quality issues—such as digital health services for vulnerable populations, data transparency, and user privacy protection—that are often overlooked in broader evaluation frameworks or alternative dimensional analyses (26). The model has been applied across diverse domains, including health care (27), transportation (28), education (29), public service (30), and e-government services (31). Owing to its multidimensional nature, SERVQUAL has been integrated with various decision-making methodologies, for example, with AHP-TOPSIS to evaluate terminal express delivery service quality (32), with EW-TOPSIS (EW-TOPSIS) to assess e-commerce-logistics service quality (33), and as the basis for an integrated fuzzy quality-function-deployment approach to measure airport service quality (34). Numerous studies applied user questionnaires to collect perceptual data on e-service quality. Alabdallat et al. (35) developed an instrument covering tangibility, reliability, responsiveness, security, and personalization. Elnaffar (36) combined an improved SERVQUAL scale with a two-dimensional mean-value matrix to visualize importance–satisfaction gaps. Although such subjective ratings reflect user experience, they are influenced by emotions, background, and sample representativeness (37), thereby introducing bias. To mitigate this subjectivity, this study employs the EW method for indicator weighting and applies the TOPSIS method to evaluate the service quality of healthcare e-government websites across Chinese cities. The EW-TOPSIS model integrates objective weighting techniques with multicriteria decision analysis and has been extensively applied in studies on digital platform openness (38), evaluations of service quality in online health communities (39), and research on the equity of medical resource allocation (40). This objective, data-driven approach not only mitigates potential biases from subjective user perceptions but also provides a scientifically sound and operationally viable reference framework. Compared with other TOPSIS methods (e.g., Neutrosophic TOPSIS (18), Fermatean Fuzzy TOPSIS (41), intervalued type-2 TOPSIS (42), q-Rung Orthopair Fuzzy TOPSIS (43)), EW-TOPSIS directly utilizes the discrete information in the government database without the need for subjective scale transformation. Moreover, the calculation is simple; it does not need for a large number of expert calibrations, high-dimensional parameter estimates, and complex defuzzification processes and is easy to explain to nontechnical decision-makers. Empirical studies on machining optimization (44) and platform governance (38) have both verified the effectiveness of the EW method in suppressing expert bias. Furthermore, the EW process is completely transparent and complies with the auditing requirements of government departments, enhancing the replicability and interpretability of this study.

Despite the methodological richness outlined above, two gaps remain. First, extant studies seldom integrate SERVQUAL with purely objective weighting schemes; most rely on expert-driven AHP or fuzzy linguistic scales, leaving room for bias when government back-office datasets are available. Second, healthcare-specific e-government evaluations lag behind both generic e-government and commercial platform research; cross-country evidence is scarce, and particularly, Chinese municipal platforms are under-represented. Addressing these gaps, this study combines SERVQUAL with EW-TOPSIS to deliver an objective, scalable assessment framework tailored to healthcare e-government.

The main contributions of this study are as follows.

(1) The novel fusion of the five-dimensional SERVQUAL model with entropy-weighted TOPSIS establishes the first sustainability-oriented evaluation system for healthcare e-government. By replacing subjective weighting with entropy-based objectivity, this framework resolves long-standing tensions between multidimensional service equity and resource efficiency, enabling life cycle assessment of digital health governance sustainability.
(2) Our multistep methodology creates an adaptive policymaking ecosystem that embeds sustainability benchmarks into digital service optimization. From dynamic indicator weighting to policy visualization, the framework institutionalizes continuous quality improvement cycles, critical for maintaining service continuity during public health crises while reducing institutional carbon footprints through data-driven resource allocation.
(3) Validated across 17 Chinese municipal platforms, this study delivers an implementation toolkit that prioritizes interoperability resilience and empathy-driven service design. This addresses the urgent need of developing nations for scalable models that balance digital infrastructure investments with human-centered sustainability outcomes, particularly in resource-constrained pandemic-recovery contexts.

## 3 Methodology

**Figure 1:**
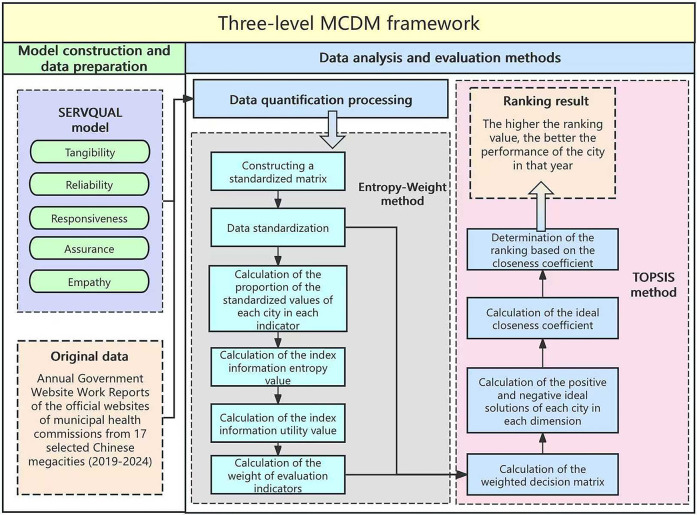
Methodological framework integrating SERVQUAL model, entropy-weight, and TOPSIS.

This study employs a three-level MCDM approach (see Figure 1). First, it integrates the five-dimensional SERVQUAL model along with corresponding subdimensions, ensuring that each subdimension is clearly defined and mutually exclusive. The five dimensions serve as the primary criteria of the decision-making model, while their respective subdimensions function as subcriteria. Second, the EW method is applied to objectively determine the weight of each dimension and subdimension. Third, the TOPSIS method is utilized to generate a ranked mapping of service quality.

### 3.1 Construction of the modified SERVQUAL model

The primary dimensions of the SERVQUAL model include tangibility, reliability, responsiveness, assurance, and empathy. Tangibility, encompassing elements such as interface design and layout, shapes users’ intuitive perceptions of website services. Reliability measures the website's ability to deliver promised services dependably, with emphasis on information accuracy and system stability. Responsiveness refers to the speed with which a company addresses user requests and inquiries and ensures timely service delivery. Assurance measures taken to build trust in data security and privacy and reliability of overall services. Finally, empathy emphasizes understanding and meeting personalized user needs, highlighting genuine care and attention.

**Figure 2:**
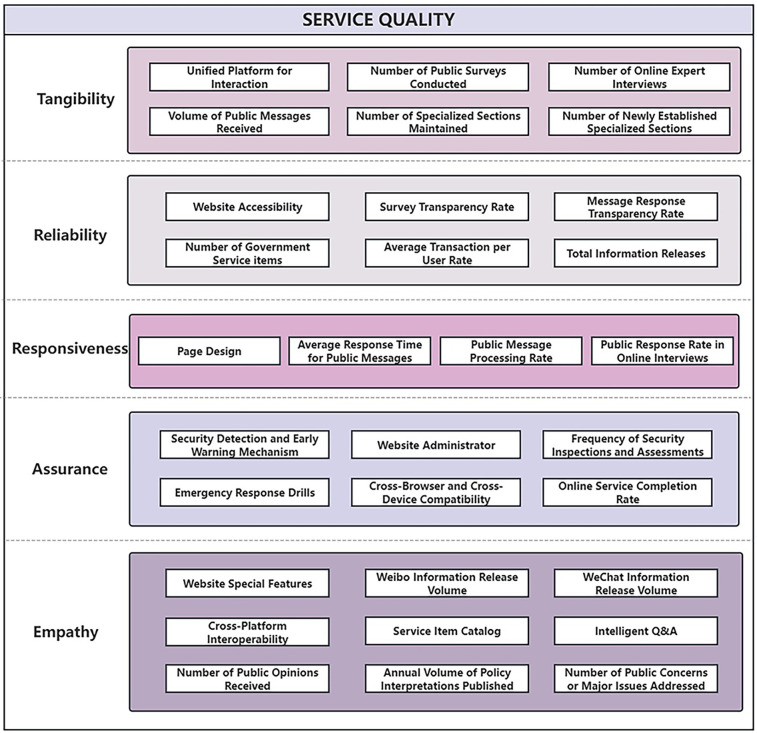
The modified SERVQUAL model.

To adapt the SERVQUAL framework to healthcare e-government websites in China, this study developed a tailored set of subdimensions based on the official evaluation indicators listed in the Annual Report on Government Websites (see Figure 2). These indicators are performance standards used by government authorities to evaluate public service websites nationwide. Based on the functional characteristics and service logic of each item, the indicators were systematically categorized into the five SERVQUAL dimensions: tangibility, reliability, responsiveness, assurance, and empathy. This classification process was done through a collaborative and iterative strategy involving multiple researchers and validation through group discussion and reviews, ensuring the internal consistency and conceptual validity of the subdimension structure.

In addition, several indicators related to the structure and user experience of government websites, such as website accessibility, page design, and cross-browser and cross-device compatibility, were included to reflect specific concerns in the online healthcare service environment. Definitions for each dimension and subdimension are provided in the Supplementary Table S1 of the Supplementary Material. All descriptions were adapted from the Annual Report on Government Websites to ensure contextual relevance and alignment with the national policy framework.

### 3.2 Data analysis and evaluation methods

Using the annual work reports published on official websites as data sources, this study quantifies the five dimensions of the SERVQUAL model, thereby reflecting the actual operational performance and service effectiveness of government websites. This study employs the EW-TOPSIS method to comprehensively evaluate the quality of municipal health commission websites.

#### 3.2.1 Selection of evaluation objects and data sources

**Figure 3:**
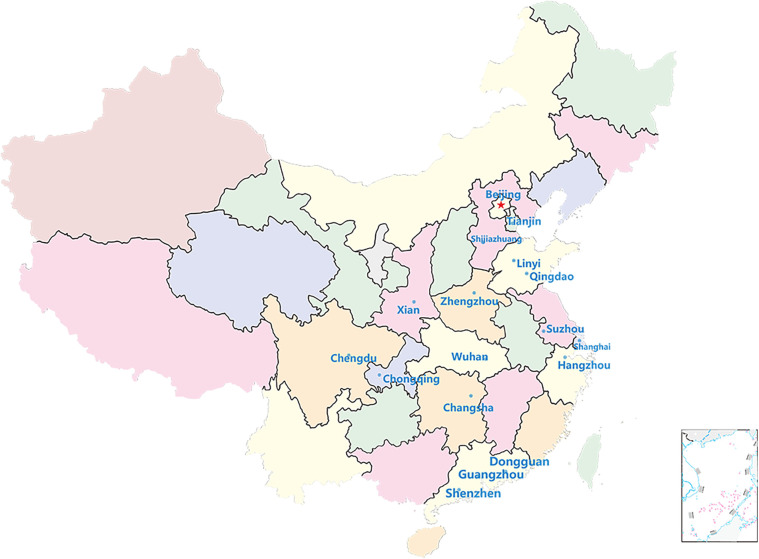
Regional distribution of 17 cities.

Herein, case data refer to official websites of municipal health commissions from 17 selected Chinese megacities, with populations of >10 million as of 2024: Beijing, Changsha, Chengdu, Chongqing, Dongguan, Guangzhou, Hangzhou, Linyi, Qingdao, Shanghai, Shenzhen, Shijiazhuang, Suzhou, Tianjin, Wuhan, Xi'an, and Zhengzhou (see Figure 3). These cities were chosen to ensure both demographic representativeness and the availability of complete multiyear data.

All data were retrieved from the Annual Government Website Work Reports published on the respective official sites. Previous studies have similarly utilized official institutional data (45, 46), which underscores the reliability and efficacy of relying exclusively on this data source. We selected these reports for four primary reasons. First, the Annual Government Website Work Reports are compiled according to uniform guidelines established by the State Council and are audited by supervisory authorities, thereby ensuring both objectivity and standardization of the data. Second, these reports encompass all 17 Chinese megacities and span the period from 2019 to 2024, effectively capturing both horizontal (intercity) and vertical (temporal) dimensions, which allows for comprehensive cross-sectional and longitudinal comparisons. Third, the Annual Government Website Work Reports are published annually and remain publicly accessible online, providing a low-cost and easily replicable data foundation for future studies. Finally, each data point can be traced back to official government websites, which enhances the transparency and credibility of the research.

The dataset includes both quantitative indicators—such as the number of public surveys conducted and the frequency of security inspections and assessments—and qualitative attributes, including emergency response drills and intelligent Q&A systems. Notably, several indicators reflecting end-user perceptions—such as page design, cross-browser and cross-device compatibility, and a unified platform—are also included, rendering the dataset relatively comprehensive. Future research may further expand and refine these perception-related indicators to enhance the evaluation framework.

To address scale heterogeneity across these diverse indicators, a unified scoring mechanism was employed. As previously outlined, binary and rule-based scoring methods were applied to qualitative features, while continuous numerical indicators were normalized using linear interpolation.

#### 3.2.2 Index weight calculated by the EW method

The EW method is an objective approach for determining indicator weights by calculating their information entropy. Information entropy reflects both the concentration of information distribution and an indicator's discriminative ability. A lower entropy value implies stronger discriminative power, thereby resulting in a greater weight and a higher impact on the overall evaluation (44). If the sample data for a particular indicator are completely identical, its influence on the overall evaluation is null, and its weight is zero.

The following formulas illustrate the entropy-based weight calculation procedure, based on the methodology outlined by Zou and Yun (47).

(1) Constructing a standardized matrix

Assume that the evaluation index system comprises *m* samples and *n* indicators. $X_{ij}$ is the value of the *j* -th evaluation index of the *i* -th sample (i=1,2,3,⋯,m;j=1,2,3,⋯,n). Based on this, a standardized matrix can be constructed, as shown in Equation 1.

(1) $$\begin{array}{c} X_{ij}=\left[\begin{array}{cccc} x_{11} & x_{12} & \cdots & x_{1n}\\ x_{21} & x_{22} & \cdots & x_{2n}\\ \vdots & \vdots & \ddots & \vdots \\ x_{m1} & x_{m2} & \cdots & x_{mn} \end{array}\right](1\leq i\leq m,0\leq j\leq n) \end{array}$$

(2) Data standardization

Because using indicators with different measurement units may introduce bias, data normalization is needed during computational processing. This step eliminates dimensional influences and generates a new data set $r_{ij}$.

(2) $$\begin{array}{c} r_{ij}=\frac{x_{ij}-min(x_{i})}{max(x_{j})-min(x_{j})} \end{array}$$

In the Equation 2, $r_{ij}$ is the value of the *j* -th evaluation index of the *i*-th sample. To ensure numerical validity, a minimal value of 0.0001 is added to $r_{ij}\;$, preventing potential computational exceptions.

(3) Calculation of the proportion y of the index value of the *i* -th object under the *j* -th index $y_{ij}$, as illustrated in Equation 3.
  (3) $$\begin{array}{c} y_{ij}=\frac{r_{ij}}{\sum_{i=1}^{m}r_{ij}}(0\leq y_{ij}\leq 1) \end{array}$$
(4) Calculation of the index information entropy value $e_{j}$, as presented in Equation 4.
  (4) $$\begin{array}{c} e_{j}=-\frac{1}{\ln m}\overset{m}{\underset{i=1}{\sum }}y_{ij}\ln y_{ij} \end{array}$$
(5) Calculation of the index information utility value $d_{j}$, as shown in Equation 5.
  (5) $$\begin{array}{c} d_{j}=1-e_{j} \end{array}$$
(6) Calculation of the weight of evaluation indicators.
  (6) $$\begin{array}{c} w_{j}=\frac{d_{j}}{\sum_{\;j=1}^{n}d_{j}} \end{array}$$

#### 3.2.3 TOPSIS method comprehensively ranks the quality of service

The TOPSIS method is widely employed in multiobjective decision analysis. As a ranking optimization technique based on similarity to an ideal solution, the TOPSIS method fully utilizes available data while minimizing the influence of subjective factors on decision outcomes. Its core principle is to identify both the ideal and negative-ideal solutions and then compute the Euclidean distance between each alternative and these reference points. An alternative that is closest to the ideal solution and farthest from the negative-ideal solution is considered the best option (48).

(1) Data standardization, see Equation 1
(2) Data normalization, see Equation 2
(3) Calculation of the weighted decision matrix $z_{ij}$.

Based on Equations 2 and 6, the weighted decision matrix is constructed using the weights derived from the EW method for each evaluation index, thereby reducing the influence of subjective factors.

(7) $$\begin{array}{c} z_{ij}=r_{ij}w_{j}(1\leq i\leq m,0\leq j\leq n) \end{array}$$

(4) Determination of the positive ideal solution $S_{j}^{+}$ and negative ideal solution $S_{j}^{-}$ of each evaluation index, according to Equations 7–9.

According to Equations 10 and 11, the Euclidean distance was used to calculate the distance between the evaluated object and the positive and negative ideal solutions $H_{i}^{+}$ $H_{i}^{-}$.

(8) $$\begin{array}{c} S_{j}^{+}=max\{z_{ij}\}(1\leq i\leq m,0\leq j\leq n) \end{array}$$

(9) $$\begin{array}{c} S_{j}^{-}=min\{z_{ij}\}(1\leq i\leq m,0\leq j\leq n) \end{array}$$

(10) $$\begin{array}{c} H_{i}^{+}=\sqrt{\overset{n}{\underset{\;j=1}{\sum }}{\left(z_{ij}-S_{j}^{+}\right)}^{2}} \end{array}$$

(11) $${H_{i}}^{-}=\sqrt{\overset{n}{\underset{\;j=1}{\sum }}{\left(z_{ij}-S_{j}^{-}\right)}^{2}}$$

(5) Calculation of the ideal closeness coefficient $c_{i}$, as defined in Equation 12.
  (12) $$\begin{array}{c} c_{i}=\frac{S_{j}^{-}}{S_{j}^{+}+S_{j}^{-}} \end{array}$$
(6) Determination of the ranking based on the closeness coefficient.

A higher closeness coefficient means superior service quality for the Official Website of the Health Commission of a city, whereas a lower value means poorer service quality.

#### 3.2.4 Data quantification processing

Because the constructed government website quality evaluation system incorporates both qualitative and quantitative data, significant differences exist in the scales and ranges of various indicators. Therefore, it is necessary to standardize these evaluation indicators quantitatively.

To ensure methodological transparency, reproducibility, and cross-city comparability, this study operationalizes 11 service quality indicators with a binary or fixed point scheme. Specifically, website accessibility, page design, and cross-browser and Cross-device compatibility are scored 100 when no performance defects are observed, and 0 otherwise; security detection and early warning mechanism, emergency response drills, website administrator, service item catalog, unified platform for interaction, and intelligent Q&A are scored 100 if the Annual Report on Government Websites confirms their presence, and 0 if absent. For the average response time for public messages, a response time of ≤3 days receives 100 and a response time of >3 days receives 0. The indicator website special features indicator is quantified at 20 points per feature (five official categories in the report), yielding 0–100 in total. Binary design is adopted because each indicator entails a clearly defined “present/absent” state, and thus, it eliminates ambiguity and simplifies the distance computation in the subsequent TOPSIS procedure. To minimize bias, the three manually tested indicators are independently appraised by multiple raters, with cross-validation and screenshot archiving to ensure consistency, while the remaining institutional indicators are taken verbatim from the authoritative government report. This scoring framework provides an objective and standardized foundation for the EW-TOPSIS evaluation. All other indicators are quantified using a value-assignment method, in which the score of each sample is determined by its relative position within the indicator range via linear interpolation. In particular, the sample value is normalized by subtracting the minimum value of the indicator, dividing by the range, and multiplying by 100 to map it on a 0–100 scale. Compared with traditional interval-based classification methods, this linear interpolation approach provides a more precise reflection of the actual distribution of sample data within the indicator range (49), thereby laying a solid foundation for the subsequent processing and computation. This method effectively resolves scale differences among various indicators, enhancing the scientific validity and rationality of the evaluation results. Table 1 presents the frequency of security inspections and assessments indicator under the assurance dimension for 2023. The middle column shows the frequency of security inspections and assessments conducted by each city in 2023, and the right column lists the assigned scores calculated using the linear interpolation method. In addition, the Calculation Example section in our Supplementary Material includes a simple calculation example of the EW-TOPSIS method, which is about the ranking calculation process of 17 cities in the tangibility dimension in 2024.

**Table 1:** Assigned value for frequency of security inspections and assessments (2023).

City	Numbers	Value
Tianjin	13	100
Beijing	12	92
Changsha	12	92
Guangzhou	12	92
Qingdao	12	92
Wuhan	12	92
Shenzhen	9	67
Xian	9	67
Chongqing	7	50
Shanghai	6	42
Chengdu	4	25
Shijiazhuang	4	25
Suzhou	4	25
Zhengzhou	4	25
Linyi	2	8
Dongguan	1	0
Hangzhou	1	0

## 4 Results

This section presents the main results from integrating the SERVQUAL model with the EW-TOPSIS method for evaluating the service quality of healthcare e-government services, as defined by the dimensions and subdimensions of the modified SERVQUAL model.

### 4.1 Weights of dimensions and subdimensions

**Figure 4:**
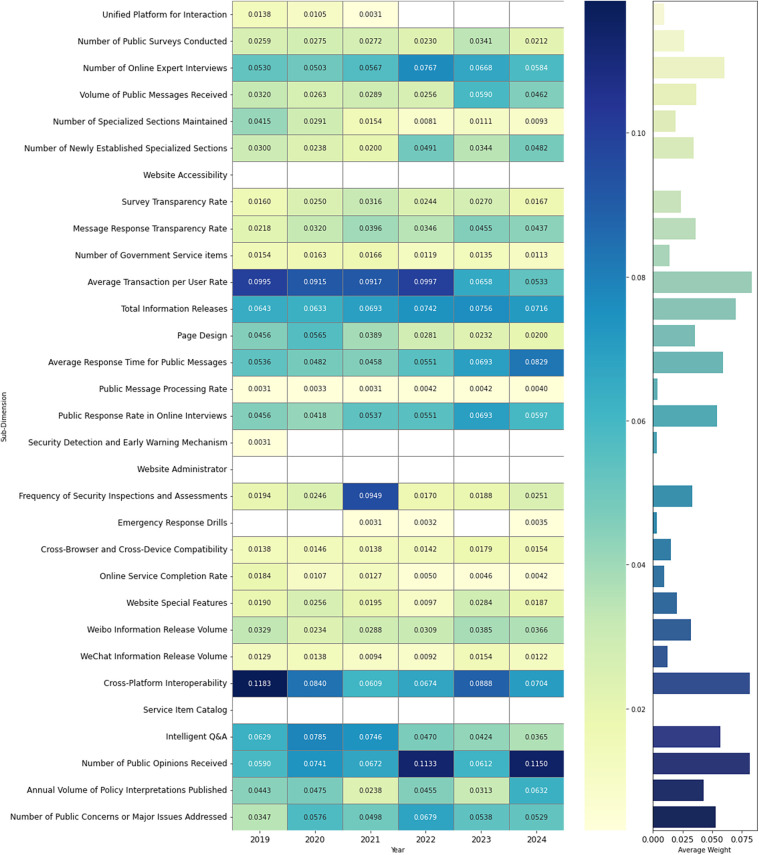
Heatmap and mean weight barplot of 31 subindicators (2019–2024).

Table 2 lists the annual weights of the five SERVQUAL dimensions from 2019 to 2024 using the EW method. A higher weight indicates greater importance in the overall evaluation. Throughout the six-year period, the empathy dimension consistently ranked first, with weights ranging from 0.3341 (2021) to 0.4054 (2024), highlighting its dominant role in user-perceived service quality. This is followed by reliability (0.2171–0.2487); tangibility, which slightly fluctuates over the years from its lowest value of 0.1513 in 2021 to its peak in 2023 at 0.2054; responsiveness (0.1415–0.1665); and, finally, assurance with notably low weights (0.0394 in 2022 and 0.0413 in 2023), indicating limited influence on the overall assessment. These overall dimension weights were calculated as the sum of the EWs of their corresponding subdimensions. By contrast, the weights of individual subdimensions varied more significantly across the years (Supplementary Table S2 in the Supplementary Material and Figure S4), where the blank part in Figure 4 indicates that the weight of this secondary dimension does not exist in the current year.

**Table 2:** Weights of dimension in 2019–2024.

Dimension	Entropy weight						Rank
	2019	2020	2021	2022	2023	2024	
Empathy	0.3842	0.4047	0.3341	0.3909	0.3598	0.4054	1
Reliability	0.2171	0.2280	0.2487	0.2448	0.2275	0.1966	2
Tangibility	0.1961	0.1676	0.1513	0.1824	0.2054	0.1833	3
Responsiveness	0.1479	0.1498	0.1415	0.1425	0.1660	0.1665	4
Assurance	0.0547	0.0499	0.1244	0.0394	0.0413	0.0481	5

### 4.2 Evaluation of healthcare e-government service quality

Evaluation was conducted based on the five dimensions of the SERVQUAL model, and comprehensive rankings were derived using the EW-TOPSIS method. The results were visualized with a ranking quality map. First, the weighted normalized matrix was calculated, as detailed in Equation 19.

#### 4.2.1 Ranking changes from 2019 to 2024

**Figure 5:**
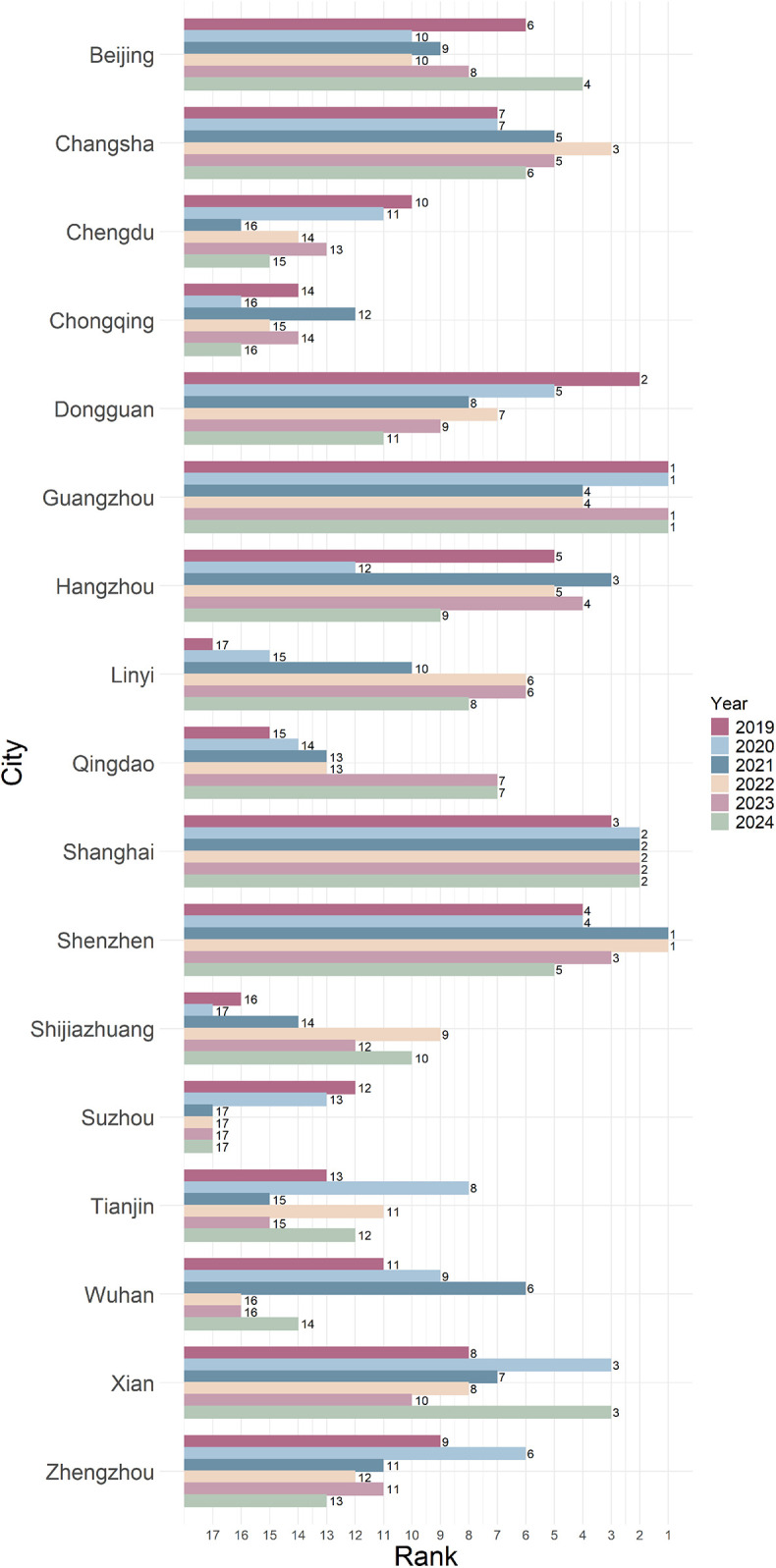
Overall ranking evolution of cities (2019–2024).

A longitudinal analysis indicates considerable heterogeneity in the service quality evaluation indices of the Official Websites of the Health Commission across cities from 2019 to 2024 (see Figure 5). The study identifies the following key characteristic.

(1) Regional disparities in service quality

The official health commission websites in economically developed regions exemplified by Shenzhen, Shanghai, and Guangzhou have consistently maintained leading positions in overall service quality assessments. Notably, Shanghai exhibits exceptional stability, ranking among the top three for six consecutive years. These findings highlight substantial regional disparities in digital governance capacity and underscore the positive correlation between investments in urban digital infrastructure and service quality outcomes.

(2) Analysis of dynamic evolution trends

The service quality index displays significant temporal fluctuations, with notable changes in city rankings. For example, Dongguan experienced a substantial decline in its comprehensive evaluation index, falling from 3rd place in 2019 to 11th place in 2024, while Zhengzhou dropped from a median ranking in 2019 to the 13th spot in 2024. By contrast, Qingdao and Linyi exhibited an upward trend in rankings. This dynamic shift suggests a zero-sum effect in intercity digital governance capabilities, whereby improvements in some cities occur at the expense of others. The longitudinal comparative study not only illustrates the evolutionary trajectory of healthcare e-government service quality but also provides empirical evidence for the differentiated development of regional digital governance capacity.

#### 4.2.2 Dynamic analysis of service quality performance

To further analyse the performance of the official health commission websites, this section systematically examines the dynamic changes in specific service quality dimensions across cities during the period of 2019–2024. This section provides a detailed explanation of the underlying factors contributing to ranking fluctuations through two analyses.

In multidimensional service quality evolution analysis, by comparing the rankings of cities across five key service quality dimensions, the unique strengths and weaknesses of the e-government practices of each region are revealed. This analysis not only offers insights into the strategic measures employed to enhance service quality but also identifies innovative models and best practices in digital governance.

In dynamic evolution analysis of the subdimensions, the changes in city rankings across various subdimensions over a six-year period are revealed, uncovering potential driving factors behind the improvement or decline in service quality. This analysis distinguishes long-term trends from short-term fluctuations, thereby facilitating an assessment of the effectiveness of policy interventions and management practices.

By integrating these two analytical approaches, this study offers a comprehensive perspective on the service quality of the Official Websites of the Health Commission, providing empirical support for city administrators to develop targeted improvement strategies and policy adjustments. This analytical framework not only deepens our understanding of the complex urban digital service environment but also supplies robust scientific evidence for enhancing public service quality (detailed data available in Supplementary Tables S3–S8 of the Supplementary Material).

##### 4.2.2.1 Multidimensional service quality evolution analysis (2019–2024)

**Figure 6:**
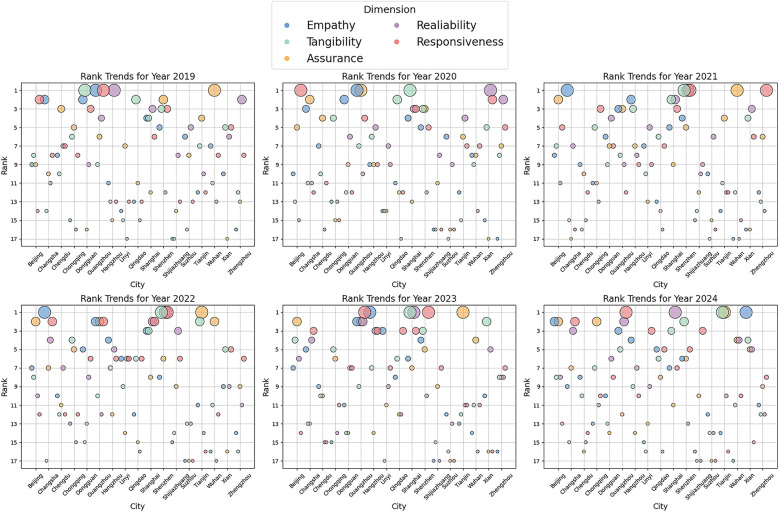
Multidimensional service quality evolution analysis (2019–2024).

This study utilizes multidimensional visualization analysis to reveal structural differences in service quality among cities, with bubble size representing the comprehensive evaluation index (see Figure 6).

(1) Advantage distribution across horizontal dimensions

  • Empathy dimension

Guangzhou has issued thousands of policy interpretations through the Guangzhou Municipal Health Commission website and developed an intelligent Q&A knowledge base to address high-frequency consultation scenarios. The optimized service architecture has considerably improved user task completion rates.

• Reliability dimension

Shanghai has enhanced its website functionality and introduced an intelligent Q&A system to improve response efficiency. Standardized message processing has resulted in a 100% on-time completion rate for public inquiries. In addition, the city has advanced the “One Thing” Reform, which streamlines government service workflows, increases user efficiency, and enhances the transparency and accessibility of e-government services.

• Tangibility dimension

Shenzhen has developed a digital healthcare public service platform that ensures 100% integration and sharing of health information resources. The promotion of the “Health Shenzhen” app, along with initiatives supporting the development of smart hospitals and smart health communities, has expanded expert and resource databases for health education. Furthermore, Shenzhen has established a regularized science popularization mechanism, organized health literacy competitions, and launched the dedicated Healthy Shenzhen Action section, thereby enhancing the intelligence of medical services and improving public health literacy.

• Responsiveness dimension

Shenzhen has optimized its website design and introduced a search function for occupational health check institutions to bolster public service accessibility. A public message big data analysis platform now accurately identifies key areas of public inquiry and complaints, facilitating efficient problem resolution.

• Assurance dimension

Tianjin has implemented a security detection and early warning mechanism, conducted regular security inspections and assessments, and organized emergency response drills. In addition, the city has optimized browser compatibility and improved the online service completion rate, ensuring both the security and operational efficiency of its government websites.

(2) Analysis of the longitudinal evolution mechanism
  •   Consistently leading type

Shenzhen has maintained a leading position in the tangibility and responsiveness dimensions through its “Triple Helix” innovation model, comprising a cloud platform, an artificial intelligence (AI) orchestration layer, and community feedback integration. In 2021, the city processed over 1,100 public requests, achieving a 100% completion rate.

• Fluctuating upward type

Tianjin has advanced its “Resilience Initiative,” resulting in a notable improvement in its assurance index—from 4th place in 2019 to 1st place in 2024. This enhancement is primarily due to a reduced response time for public health early warnings, which has facilitated a more accurate and timely warning mechanism.

• Phase-declining type

Due to fragmentation within its digital governance system, Zhengzhou experienced a decline in its responsiveness ranking, falling from 1st place in 2021 to 8th place in 2024, accompanied by a slight deterioration in website response capability.

(3) Innovation-driven factors
  •   Technology empowerment

Shanghai's “One Network for Integrated Governance” system has significantly enhanced government response speed since 2020.

• Institutional innovation

Changsha launched China's first pilot project to standardize e-government services in 2019.

• Public health service ecosystem

Hangzhou introduced several innovative initiatives in 2023, including the Trusted Cloud-Based Herbal Medicine Processing e-Platform, AI-enhanced medical escort service, and health brain smart framework.

This multidimensional analytical framework effectively deconstructs the dynamic formation mechanisms underlying digital government service quality, providing a verifiable evolutionary model for smart city governance. Empirical data indicate that cities implementing digital transformation strategies have experienced a year-on-year increase in their service efficiency index, significantly outperforming those reliant on traditional governance models.

##### 4.2.2.2 Dynamic evolution analysis of subdimensions (2019–2024)

**Figure 7:**
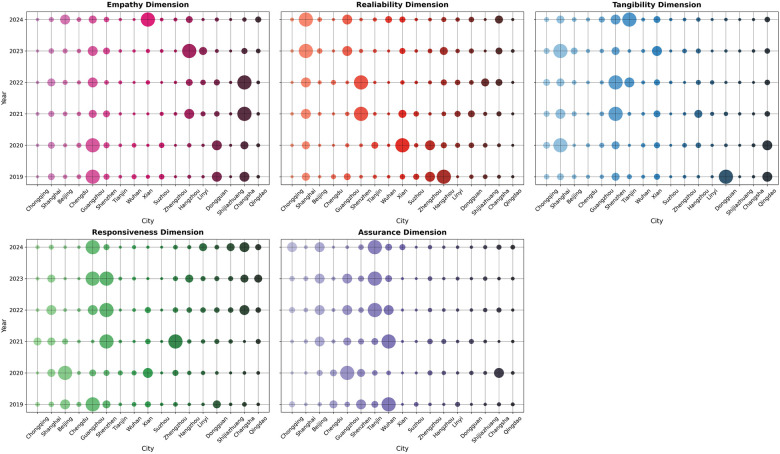
Dynamic evolution analysis of subdimensions (2019–2024).

This study conducts a detailed analysis of fluctuations in the subdimensions of healthcare e-government service quality across cities in the period of 2019–2024. These fluctuations reveal the underlying factors by which subdimensions influence overall dimensions and cause ranking changes while illustrating the improvement measures implemented by cities to enhance service quality (see Figure 7).

• Empathy dimension

Guangzhou has consistently led in the cross-platform interoperability, service item catalog, and intelligent Q&A indicators. By posting health education information on its official website and organizing events such as the “Health Cup” Competition, the city has strengthened public health awareness. However, its relative shortcomings in other indicators indicate that there is still room for overall improvement in the empathy dimension. Beijing has significantly enhanced information transparency and public satisfaction by implementing multichannel policy interpretation, improving its immediate complaint resolution mechanism, strengthening public opinion monitoring and emergency response, and increasing public interaction and engagement. These efforts have contributed to the continuous healthcare services optimization. Conversely, Dongguan experienced a decline in ranking due to weaker performance in indicators such as the annual volume of policy interpretations published, which highlights the importance of timely response to public concerns in service quality evaluation.

• Reliability dimension

Shanghai has demonstrated continuous improvement in survey transparency rate and average transaction per user rate, thereby enhancing information disclosure through the online service completion platform. By contrast, Shenzhen and Xi'an have experienced notable fluctuations in the average transaction per user rate, constrained by service frequency and system performance instability, which negatively impacted user experience and satisfaction.

• Tangibility dimension

Shanghai and Shenzhen have consistently maintained their leading positions, with both cities achieving full scores on the unified platform for interaction and demonstrating exceptional cross-departmental collaboration capabilities. In addition, Shenzhen has adopted a proactive approach to public demand responsiveness and information interpretation by introducing new specialized sections on its platform.

• Responsiveness dimension

Shenzhen has maintained a high level of stability by optimizing page design and service responsiveness. By contrast, Beijing experienced a significant decline, with sharp drops in the average response time for public messages and the public response rate in online interviews, leading to a severe rank decrease. Guangzhou has shown short-term fluctuations, indicating that insufficient capacity for processing user feedback has adversely affected its performance in this dimension.

• Assurance dimension:

Beijing and Tianjin have achieved significant improvements in the frequency of security inspections and assessments and the online service completion rate, thereby enhancing platform accessibility and convenience. Conversely, Wuhan has presented score fluctuations in these indicators, indicating insufficient stability in its security assurance capabilities.

Therefore, the continuous development and improvement across different subdimensions have significantly influenced the overall service quality ranking of each city. Through a longitudinal interpretation of comprehensive data, this study provides empirical support for government agencies in formulating and optimizing e-government service strategies. This further recommends prioritizing long-term stability and comprehensive capability enhancement to achieve an efficient and sustainable Official Website of the Health Commission service system.

## 5 Discussion

### 5.1 Analysis of imbalances in the development of healthcare e-government services across cities

#### 5.1.1 Impact of economic development

This study reveals a notable moderating effect of regional economic heterogeneity on the development of healthcare e-government services. Economic development acts as a catalyst of adoption and expansion of e-government services, particularly in large cities with populations of >10 million, where economic disparities substantially impact the progress and quality of healthcare e-government services (2, 50).

For example, Shenzhen has demonstrated a leading position in healthcare e-government services by leveraging robust financial support and policy advantages. According to the TOPSIS-based ranking results, Shenzhen remained within the top five cities for six consecutive years and ranked first in both 2021 and 2022, reflecting its consistent excellence and dominant performance in service quality. Its strong industrial foundation and vibrant innovation ecosystem, particularly in AI and big data, provide solid technological backing and financial guarantees for the development of smart healthcare and healthcare e-government platforms. This combination of technology and funding enables Shenzhen to deliver high-quality e-government services under uniquely favorable conditions. By contrast, cities with depressed economies face challenges in building healthcare e-government services due to funding shortages and uneven resource allocation. These cities often lack industrial and technological research capabilities necessary to support the growth of high-end industries, thereby limiting their progress in smart healthcare and data integration compared to cities with robust economies. Although basic functionalities have been deployed, challenges in optimizing user experience and improving response efficiency persist due to technological and resource constraints, hindering comprehensive service quality enhancement.

#### 5.1.2 Relevance of regional information technology infrastructure

This study examines key factors, such as technological infrastructure levels, professional talent reserves, and data interoperability, and explores their impact on the uneven development of urban e-government services (51). Cities with strong technological resources often establish highly efficient network environments, modernized data centers, and advanced digital platforms, thereby providing a solid foundation for the effective operation of digital healthcare services. By contrast, cities with limited technological resources may contend with outdated infrastructure and insufficient technical support, which restrict the functionality of their platforms.

The case of Shanghai illustrates how technological advantages contribute to improved e-government service efficiency. Shanghai is not only a domestic leader in AI development but also excels in data governance and infrastructure construction. This is reflected in its strong performance in the reliability and tangibility dimensions, where it ranked within the top three cities in most years between 2019 and 2024. In particular, the reliability scores of Shanghai improved from 3rd place in 2019 and 2020 to 1st place in both 2023 and 2024. Meanwhile, it ranked 1st place in the tangibility dimension in 2020 and 2023 and never fell below 4th place. These consistent high rankings position Shanghai as a national benchmark for digital governance in healthcare services. By establishing a comprehensive health data platform, the city has achieved centralized data management and utilization. Systems such as “One-Code Medical Service” and mobile health service platform have significantly streamlined medical service processes. Furthermore, with strong computational power and robust industry–academia–research collaboration, Shanghai has become a hub of technological innovation while providing a replicable model for e-governance.

By contrast, some cities lack modernized data governance mechanisms, limiting their ability to integrate and share health data. This deficiency results in inefficient, redundant service processes that detrimentally impact overall efficiency and effectiveness. Additionally, slow response times and poor system stability of the e-government platforms of these cities contribute to a suboptimal user experience, reducing public engagement and the willingness to use e-government services.

#### 5.1.3 Policy-driven development and urban disparities

This study examines progress of China in e-government initiatives and the developmental imbalances among its cities. The analysis indicates that national-level initiatives, such as the “Internet plus” policy and 5G investments, have succeeded in enhancing service delivery and modernizing public administration (52). For example, the Action Plan for the Integrated Development of Virtual Reality and Industrial Applications (2022–2026) demonstrates a strong commitment to virtual technology applications, while Jointly Build a Community with a Shared Future in Cyberspace underscores the importance of cooperation in cybersecurity with countries around the world.

However, disparities in policy support and strategic implementation among cities have emerged as a major factor contributing to imbalances in e-government development. In particular, in Guangzhou, strategic planning and policy orientation have provided a solid foundation for the efficient implementation of healthcare e-government services. The 14th Five-Year Plan for Guangzhou Municipal Health Development has promoted standardized development in three key areas of health informatization: regional health, hospitals, and public health (53). Through the implementation of the “Internet plus Healthcare” model, Guangzhou has achieved full coverage of electronic health codes, significantly improving service accessibility and efficiency. This progress is also reflected in the evaluation results: Guangzhou consistently ranked among the top three cities in the empathy dimension from 2019 to 2024, highlighting its strong performance in delivering patient-centered and user-responsive services.

By contrast, some cities lack clear policy objectives and long-term development plans, resulting in uncertain technology adoption and resource allocation that limit service coverage and infrastructure development. These cities lag in both the depth and breadth of e-government services, failing to adequately address diverse public needs. Moreover, weak policy execution negatively impacts user experience and compromises data integration optimization.

#### 5.1.4 Digital accessibility and public demand disparities

This study examines the impact of differences in public demand and uneven digital accessibility (54). In economically developed cities such as Shanghai, Beijing, Guangzhou, and Shenzhen, well-established digital infrastructure and high levels of digital literacy fuel strong demand for efficient and personalized e-government services. For example, Shanghai has implemented cross-departmental data sharing via a big data platform to fulfill resident expectations for smart services. Similarly, Shenzhen has successfully integrated electronic health records with medical services through its “Internet plus Healthcare” initiative. The long-term leading positions of these cities in the empathy dimension also reflect their strengths in user-centered interaction and feedback mechanisms. By contrast, cities such as Linyi and Shijiazhuang, despite having large populations, exhibit relatively lower demand for online services due to weaker economic foundations and limited digital capabilities. From 2019 to 2024, these cities consistently ranked lower in the empathy dimension, particularly in the subdimensions cross-platform interoperability and intelligent Q&A, indicating persistent gaps in seamless interaction and timely response. In these cities, constrained digital skills restrict online service usage, resulting in e-government platforms that primarily support basic functions and fall short of meeting advanced service needs.

The imbalance in digital accessibility is particularly pronounced in the allocation of technological resources and the promotion of digital literacy. For example, Beijing and Wuhan have made significant investments in educational resources and technology training, successfully enhancing the digital capabilities of their citizens. By contrast, other cities lag in network infrastructure development, thereby creating a digital accessibility divide between urban and rural areas. For example, Suzhou and Zhengzhou experience unstable network signals in urban–rural transition zones, which negatively affect both the accessibility and user experience of e-government services.

### 5.2 Recommendations on sustainable development strategies

This study demonstrates that empathy and reliability are the key dimensions of healthcare e-government services and deserve the priority of regulatory authorities. The public's needs shifted markedly after the COVID-19 pandemic, making these dimensions especially critical in AI- and data integration–driven e-government services (55). The pandemic has served as a pivotal turning point, transitioning service demands from basic information access to intelligent interaction (56). Based on multidimensional evaluation results, this study proposes the following systematic optimization recommendations.

#### 5.2.1 Empathy enhancement strategies

Empathy has emerged as a core dimension in the evaluation of healthcare e-government services. A closer examination of subdimension weights from 2019 to 2024 reveals that cross-platform interoperability, intelligent Q&A, and number of public opinions received consistently contributed the most to this dimension. Notably, cross-platform interoperability ranked first in every year, peaking at 0.1183 in 2019. Both intelligent Q&A and number of public opinions received also maintained relatively high and stable weights throughout the period. These findings underscore the public's growing expectations for seamless cross-platform interaction, timely government responses, and accessible feedback mechanisms.

This pattern aligns with post-pandemic digital behavior trends where users increasingly engage through mobile apps, web portals, and social media platforms, expecting integrated and timely service delivery. The high weight of cross-platform interoperability, often overlooked in traditional offline-centric service evaluations, reflects a fundamental shift toward seamless, platform-neutral service delivery. Similarly, intelligent Q&A systems are seen as essential for improving perceived responsiveness and alleviating the burden on human staff, particularly in times of public health emergencies. These results reinforce the notion that empathy in e-government is no longer limited to passive information delivery but hinges on active, intelligent, and participatory interaction, which is in line with contemporary governance expectations.

To address these critical needs, the following strategies are proposed. First, a multichannel information diffusion matrix should be established to facilitate empathetic communication and interaction (57). This involves enhancing intelligent Q&A systems by deploying natural language processing–based chatbots, for example, Spain's Hispabot-Covid19 model, which effectively answered over 200 pandemic-related queries (58). Second, digital inclusion must be prioritized by setting up smart health kiosks in underserved areas, integrating features such as free Wi-Fi and multilingual service interfaces to bridge digital divides. Third, interactive policy coordination can be improved by employing social media sentiment analysis to build a dynamic demand-policy alignment model, thereby allowing government to better meet evolving public expectations (59). Together, these strategies directly target the most influential empathy subdimensions and support the creation of a more inclusive, responsive, and citizen-centered healthcare e-government environment, offering concrete paths for cities to enhance perceived service quality where it matters most.

#### 5.2.2 Reliability optimization pathway

A detailed analysis of subindicator weights from 2019 to 2024 highlights average transaction per user rate and total information releases as the two most influential indicators within the reliability dimension. Notably, the average transaction per user rate indicator consistently held the highest weight among all subindicators for multiple years, with values of >0.09 in most cases. The total information releases indicator also maintained a stable and relatively high contribution, particularly between 2021 and 2024, reflecting user expectations for platform efficiency and transparency.

To improve reliability, the following strategies are proposed. First, user engagement should be enhanced by analyzing transaction data to optimize high-frequency service functions, and personalized behavioral models should be developed (60, 61). Second, unified service delivery should be promoted by advancing the online service completion strategy and establishing a coordinated, interdepartmental processing mechanism. Third, services should be inclusive by developing intelligent assistance tools for special user groups, thereby reducing digital exclusion and promoting broader access to dependable e-government healthcare services (62).

#### 5.2.3 Tangibility enhancement strategies

Interview platforms serve as channels for both policy dissemination and interpretation while fostering public engagement in decision-making and enhancing trust between the government and its citizens. Strategies to improve interview efficiency include establishing an interactive mechanism by systematically conducting government–public interviews and creating a closed-loop system for demand collection, analysis, and response (63) and developing multidimensional feedback channels through online surveys, digital questionnaires, and other digital tools (64).

#### 5.2.4 Responsiveness enhancement framework

Average response time for public messages is a key indicator for assessing the efficiency of e-government services and bolstering public trust. Timely responses enable users to quickly access relevant information and promote transparency in government information disclosure. To enhance responsiveness, the following measures are recommended: (a) to use an intelligent response system, a hybrid service model can be deployed, which integrates AI-powered customer service with dedicated human support teams (65, 66); and (b) for a multilevel processing mechanism, a classification-based transfer and tracking feedback system can be established to handle complex inquiries (67, 68).

#### 5.2.5 Assurance enhancement plan

Within the assurance dimension, security inspections and assessments are paramount. Therefore, efforts should prioritize the following areas: (a) security protection upgrades by establishing a multilayer security architecture to enhance data encryption and access control (69, 70), (b) an intelligent monitoring system by implementing AI-driven real-time security monitoring (71, 72), and (c) an emergency response mechanism by forming specialized emergency response teams to ensure continuous service provision (73).

## 6 Conclusions

This study constructs a comprehensive evaluation framework that integrates the SERVQUAL model, EW method, and TOPSIS method to assess the quality of healthcare e-government services in 17 Chinese megacities. The proposed model enables a multidimensional, data-driven analysis and yields the following key findings:

Empathy emerges as the most influential dimension across all years, with Cross-platform interoperability, intelligent Q&A, and number of public opinions received consistently ranking among the top contributors. Despite traditionally being viewed as a “soft” dimension, our results highlight its central role in fostering user-centered, interactive government services. Similarly, reliability is strongly influenced by average transaction per user rate and total information releases, while tangibility is mainly driven by the number of online expert interviews. Responsiveness hinges on average response time for public messages, and assurance relies on security inspections and assessments. These findings provide a structured understanding of user priorities and inform the optimization of e-government interfaces.

The cross-city ranking results reveal substantial service quality gaps, indicating that disparities in economic capacity, technological resources, and administrative leadership remain key obstacles to balanced development. Some cities consistently lag in core dimensions such as empathy and responsiveness, pointing to systemic shortcomings in user-centered design and timely response mechanisms. However, these differences also present an opportunity for collaborative benchmarking and knowledge transfer between cities, thereby transforming regional imbalances into catalysts for policy learning and governance innovation. This perspective aligns with Sustainable Development Goal (SDG) 10 (Reduced Inequalities) by advocating targeted interventions in underperforming regions and promoting more equitable access to digital health services across urban populations.

This study demonstrates both methodological innovation and practical value. The integration of the SERVQUAL–EW-TOPSIS model with a binary/fixed value scoring strategy significantly enhances the consistency and interpretability of evaluation results. This approach minimizes subjective bias and provides a stable, replicable evaluation pathway, especially suitable for multiregion, multi-indicator comparisons in complex governance environments. At the practical level, the proposed framework supports a closed-loop policy optimization mechanism, enabling governments to identify weak points, prioritize interventions, and monitor improvement outcomes over time. It also provides a scalable decision-support tool for other public sectors and is especially valuable for low- and middle-income countries striving to improve digital healthcare governance. These contributions directly serve SDG 3 (Good Health and Well-being) by addressing health service gaps, support SDG 10 (Reduced Inequalities) through fairer access mechanisms, and advance SDG 11 (Sustainable Cities and Communities) by enhancing inclusive and resilient digital public services.

## 7 Limitations

This study encounters several limitations that warrant further investigation. First and foremost, while the SERVQUAL model provides a valuable framework for assessing service quality, it does not fully encompass critical technical dimensions such as website performance and user data security. This oversight may potentially limit the comprehensiveness of the service quality evaluation. Furthermore, the study has focused solely on 17 megacities, which constrains the generalizability of the findings to small- and medium-sized cities as well as other regions. Additionally, although the EW-TOPSIS method offers objectivity in weight allocation, its heavy reliance on data dispersion may not accurately reflect user preferences.

To address these limitations, future research should aim to integrate additional technical indicators and broaden the evaluation scope to encompass smaller municipalities and various administrative contexts. This approach would facilitate the exploration of service quality improvement strategies tailored to regional characteristics. Moreover, we recommend adopting a mixed-methods design that merges the current institutional-data-based framework with user-centered data collection strategies. These strategies might include large-scale online surveys, focus group interviews, and citizen feedback platforms. Notably, several indicators within the existing framework—such as page design, cross-browser and cross-device compatibility, and unified platforms for interaction—already reflect user-facing aspects to some degree. These indicators could be further validated and expanded through empirical user feedback, leading to a more comprehensive understanding of user needs.

This mixed-methods approach would enable the inclusion of user-derived indicators within the SERVQUAL hierarchy and support the development of hybrid weighting strategies that effectively balance objective performance with subjective user experience. Furthermore, future studies could investigate the association between quality scores and real-world outcomes, such as user satisfaction, digital health literacy improvements, and service utilization rates. Such investigations would contribute to the empirical validation of the practical impact of service quality and significantly enhance both the comprehensiveness and practical value of this study.

## Data Availability

The raw data supporting the conclusions of this article will be made available by the authors, without undue reservation.
