# Supplementary material for: Sustainable service quality assessment of Chinese healthcare e-government: a multi-criteria decision framework based on SERVQUAL model and entropy-weight TOPSIS method
Source: Front Digit Health. 2025 Sep 8;7:1611979. doi: 10.3389/fdgth.2025.1611979 (PMC12450976; doi:10.3389/fdgth.2025.1611979)
Supplement: Supplementary file 1 [file Datasheet1.pdf]

Supplementary Material

1 Supplementary Data

1.1 Supplementary Tables

Supplementary Table 1 Components of the modified SERVQUAL model

Dimension	Subdimension	Description
Tangibility	Unified Platform for Interaction	The implementation of interactive communication integration within the unified platform for interaction directly affects the users' ability to seamlessly access and utilize various services.
	Number of Public Surveys Conducted	This indicator reflects the frequency with which public opinions and demands are collected on the website. Moreover, effective physical presentation and design can significantly enhance user engagement.
	Number of Online Expert Interviews	The number of online expert interviews organized by the website reflects its capacity for online interaction and public communication, serving as an indicator of platform tangibility.
	Volume of Public Messages Received	The volume of public messages received directly reflects user interaction and engagement on the website, illustrating its effectiveness as a communication platform.
	Number of Specialized Sections Maintained	The number of specialized sections maintained on the website reflects its update frequency and content quality.
	Number of Newly Established Specialized Sections	The number of newly established specialized sections reflects the website's substantive investment in delivering updated information and innovative features.
Reliability	Website Accessibility	Website accessibility is the most fundamental performance requirement, directly determining whether users can effectively access the site. It is a key aspect of service reliability.
	Survey Transparency Rate	The survey transparency rate reflects the consistency of information disclosure on government websites, ensuring that users have access to reliable survey feedback and thereby enhancing service stability.
	Message Response Transparency Rate	This indicator reflects the transparency of feedback processing on government websites, ensuring that users consistently have access to authentic responses and thereby enhancing the credibility of website services.

Dimension	Subdimension	Description
Responsiveness	Number of Government Service items	This metric measures both the number of services offered by the website and their breadth of coverage. A higher number of service items indicates a stronger service delivery capability.
	Average Transaction per User Rate	This measure assesses how efficiently users complete transactions, thereby reflecting the actual reliability of the service.
	Total Information Releases	The volume of total information releases directly reflects the website's capacity to deliver timely and accurate information services.
	Page Design	Page design directly influences user experience; its conciseness and visual appeal affect the users' ability to quickly locate the information that they need.
	Average Response Time for Public Messages	This measure is a direct indicator of the website's response speed. The capacity to respond swiftly to user feedback demonstrates the website's responsiveness.
	Public Message Processing Rate	The indicator measures the website's response speed and problem-solving capabilities.
	Public Response Rate in Online Interviews	This measure quantifies the website's responsiveness by measuring its replies to citizens' questions during online interviews.
Assurance	Security Detection and Early Warning Mechanism	The indicator ensures that appropriate protective measures are implemented when the website encounters security threats, thereby instilling a sense of security in users.
	Website Administrator	Having a website administrator signifies a robust management mechanism, which in turn enhances user trust in the website's services.
	Frequency of Security Inspections and Assessments	The frequency of security inspections and assessments ensures the technical security of the website, thereby enhancing user trust and instilling confidence in the platform's safety and reliability.
	Emergency Response Drills	This indicator demonstrates the website's capability to manage emergencies, thereby enhancing user trust in service stability and bolstering their sense of security.
	Cross-Browser and Cross-Device Compatibility	Cross-browser and cross-device compatibility ensures that the website functions seamlessly across mainstream browsers and devices, enabling users to access all services without technical limitations. This capability enhances user trust and reinforces their sense of security while using the website.

Dimension	Subdimension	Description
Empathy	Online Service Completion Rate	The online service completion rate reflects the government website's commitment to service integration and process simplification, ensuring that users can complete transactions efficiently. Consequently, this metric reinforces user trust in both the reliability and assurance of the website’s services.
	Website Special Features	The website’s special features, developed by the government to meet user demands or enhance website usage, exemplify its commitment to carefully addressing users’ specific needs.
	Weibo Information Release Volume	Disseminating information via Weibo aligns more closely with users’ information consumption habits.
	WeChat Information Release Volume	Offering information release services via WeChat demonstrates the website’s commitment to addressing the diverse needs of its users.
	Cross-Platform Interoperability	The government’s capacity to disseminate information via other applications provides users with more convenient access channels.
	Service Item Catalog	Publishing the Service Item Catalog facilitates user inquiries and demonstrates the government’s commitment to delivering personalized, convenient services.
	Intelligent Q&A	The intelligent Q&A function assists users in swiftly and efficiently resolving personalized issues, thereby reflecting the website’s empathetic orientation.
	Number of Public Opinions Received	The process of collecting public opinions reflects the website’s attentiveness to user needs in service delivery and its commitment to providing personalized services.
	Annual Volume of Policy Interpretations Published	The number of policy interpretation releases indicates the level of support provided by the government website to help users understand policies, thereby demonstrating its commitment to addressing user needs.
	Number of Public Concerns or Major Issues Addressed	The number of public concerns or major issues addressed reflects the government's commitment to promptly addressing public concerns and providing timely responses, thereby demonstrating its conscientious attention to users.

Supplementary Table 2 Weights of subdimension in 2019-2024

Dimension	Subdimension	2019	2020	2021	2022	2023	2024
		Entropy weight	Entropy weight	Entropy weight	Entropy weight	Entropy weight	Entropy weight
Tangibility	Unified Platform for Interaction	0.0138	0.0105	0.0031	-	-	-
	Number of Public Surveys Conducted	0.0259	0.0275	0.0272	0.0230	0.0341	0.0212
	Number of Online Expert Interviews	0.0530	0.0503	0.0567	0.0767	0.0668	0.0584
	Volume of Public Messages Received	0.0320	0.0263	0.0289	0.0256	0.0590	0.0462
	Number of Specialized Sections Maintained	0.0415	0.0291	0.0154	0.0081	0.0111	0.0093
	Number of Newly Established Specialized Sections	0.0300	0.0238	0.0200	0.0491	0.0344	0.0482
Reliability	Website Accessibility	-	-	-	-	-	-
	Survey Transparency Rate	0.0160	0.0250	0.0316	0.0244	0.0270	0.0167
	Message Response Transparency Rate	0.0218	0.0320	0.0396	0.0346	0.0455	0.0437
	Number of Government Service items	0.0154	0.0163	0.0166	0.0119	0.0135	0.0113
	Average Transaction per User Rate	0.0995	0.0915	0.0917	0.0997	0.0658	0.0533
Responsiveness	Total Information Releases	0.0643	0.0633	0.0693	0.0742	0.0756	0.0716
	Page Design	0.0456	0.0565	0.0389	0.0281	0.0232	0.0200
	Average Response Time for Public Messages	0.0536	0.0482	0.0458	0.0551	0.0693	0.0829
	Public Message Processing Rate	0.0031	0.0033	0.0031	0.0042	0.0042	0.0040
	Public Response Rate in Online Interviews	0.0456	0.0418	0.0537	0.0551	0.0693	0.0597
Assurance	Security Detection and Early Warning Mechanism	0.0031	-	-	-	-	-
	Website Administrator	-	-	-	-	-	-
	Frequency of Security Inspections and Assessments	0.0194	0.0246	0.0949	0.0170	0.0188	0.0251
	Emergency Response Drills	-	-	0.0031	0.0032	-	0.0035
	Cross-Browser and Cross-Device Compatibility	0.0138	0.0146	0.0138	0.0142	0.0179	0.0154
Empathy	Online Service Completion Rate	0.0184	0.0107	0.0127	0.0050	0.0046	0.0042
	Website Special Features	0.0190	0.0256	0.0195	0.0097	0.0284	0.0187
	Weibo Information Release Volume	0.0329	0.0234	0.0288	0.0309	0.0385	0.0366
	WeChat Information Release Volume	0.0129	0.0138	0.0094	0.0092	0.0154	0.0122
	Cross-Platform Interoperability	0.1183	0.0840	0.0609	0.0674	0.0888	0.0704
	Service Item Catalog	-	-	-	-	-	-
	Intelligent Q&A	0.0629	0.0785	0.0746	0.0470	0.0424	0.0365
	Number of Public Opinions Received	0.0590	0.0741	0.0672	0.1133	0.0612	0.1150
	Annual Volume of Policy Interpretations Published	0.0443	0.0475	0.0238	0.0455	0.0313	0.0632
	Number of Public Concerns or Major Issues Addressed	0.0347	0.0576	0.0498	0.0679	0.0538	0.0529

Supplementary Table 3 Value of each city in 2019

Dimension	Sub-dimension	Chongqing	Shanghai	Beijing	Chengdu	Guangzhou	Shenzhen	Tianjin	Wuhan	Xian	Suzhou	Zhengzhou	Hangzhou	Linyi	Dongguan	Shijiazhuang	Changsha	Qingdao
	Unified Platform for Interaction	100	100	0	100	100	100	100	100	100	0	100	0	100	100	0	100	100
	Number of Public Surveys Conducted	59	15	22	7	63	52	7	15	11	11	22	11	0	100	11	11	0
	Number of Online Expert Interviews	9	55	2	0	6	9	4	17	4	0	9	2	0	100	0	0	28
Tangibility	Volume of Public Messages Received	47	27	33	1	11	40	8	5	100	6	11	18	0	1	5	3	12
	Number of Specialized Sections Maintained	7	8	9	3	8	22	6	4	8	0	3	10	2	44	8	1	100
	Number of Newly Established Specialized Sections	0	0	75	50	25	100	75	25	0	25	25	50	0	25	0	0	25
	Website Accessibility	100	100	100	100	100	100	100	100	100	100	100	100	100	100	100	100	100
	Survey Transparency Rate	0	25	0	100	100	100	100	100	100	100	100	100	0	48	100	100	0
Reliability	Message Response Transparency Rate	4	21	11	100	8	8	79	4	99	100	4	83	0	100	100	72	68
	Number of Government Service items	90	25	100	7	78	72	6	70	48	67	61	52	32	83	0	0	20
	Average Transaction per User Rate	0	3	1	0	6	0	0	0	0	0	55	100	0	0	0	0	0
	Total Information Releases	4	100	15	8	33	1	2	1	2	1	1	4	2	0	1	6	1
	Page Design	0	100	100	0	100	0	100	100	100	0	0	0	0	0	0	100	0
Responsiveness	Average Response Time for Public Messages	0	0	100	100	100	100	0	0	100	0	0	0	0	100	0	0	0
	Public Message Processing Rate	100	100	98	100	100	100	0	100	97	100	100	100	100	100	100	100	100
	Public Response Rate in Online Interviews	100	100	100	0	100	100	0	0	0	0	100	0	0	100	0	0	0

Dimension	Sub-dimension	Chongqing	Shanghai	Beijing	Chengdu	Guangzhou	Shenzhen	Tianjin	Wuhan	Xian	Suzhou	Zhengzhou	Hangzhou	Linyi	Dongguan	Shijiazhuang	Changsha	Qingdao
Assurance	Security Detection and Early Warning Mechanism	100	100	100	100	100	100	100	100	100	100	100	100	100	100	0	100	100
	Website Administrator	100	100	100	100	100	100	100	100	100	100	100	100	100	100	100	100	100
	Frequency of Security Inspections and Assessments	10	30	20	60	5	40	15	80	25	30	20	20	0	10	5	100	20
	Emergency Response Drills	100	100	100	100	100	100	100	100	100	100	100	100	100	100	100	100	100
	Cross-Browser and Cross-Device Compatibility	100	100	100	100	100	100	100	100	0	0	100	100	100	100	100	0	0
Empathy	Online Service Completion Rate	95	10	54	41	99	97	100	100	0	100	19	1	100	4	32	6	78
	Website Special Features	20	60	40	0	60	20	0	20	20	20	20	60	40	40	0	40	0
	Weibo Information Release Volume	11	26	2	100	24	48	57	1	83	6	0	27	1	2	13	0	29
	WeChat Information Release Volume	54	50	2	88	54	48	56	0	98	28	84	34	34	18	34	11	100
	Cross-Platform Interoperability	0	0	0	0	100	0	0	28	0	0	0	0	0	0	0	0	0
	Service Item Catalog	100	100	100	100	100	100	100	100	100	100	100	100	100	100	100	100	100
	Intelligent Q&A	0	100	0	0	100	100	0	0	0	0	0	0	0	100	0	100	0
	Number of Public Opinions Received	4	2	44	0	12	10	0	0	4	100	2	4	0	4	2	39	0
	Annual Volume of Policy Interpretations Published	16	10	4	4	16	6	1	1	3	30	1	10	0	100	5	18	2
	Number of Public Concerns or Major Issues Addressed	1	37	31	4	37	31	0	0	0	39	0	16	1	100	15	67	9

Supplementary Table 4 Value of each city in 2020

Dimension	Sub-dimension	Chongqing	Shanghai	Beijing	Chengdu	Guangzhou	Shenzhen	Tianjin	Wuhan	Xian	Suzhou	Zhengzhou	Hangzhou	Linyi	Dongguan	Shijiazhuang	Changsha	Qingdao
Tangibility	Unified Platform for Interaction	100	100	100	100	100	100	100	0	100	0	100	100	100	100	0	100	100
	Number of Public Surveys Conducted	100	30	22	22	26	74	4	15	4	7	19	26	0	15	11	11	0
	Number of Online Expert Interviews	14	100	0	0	14	41	0	27	0	0	14	14	0	5	0	5	59
	Volume of Public Messages Received	66	100	31	15	29	23	7	0	90	8	15	37	5	4	9	6	25
	Number of Specialized Sections Maintained	19	12	5	18	13	18	7	6	8	0	10	10	5	37	11	7	100
	Number of Newly Established Specialized Sections	100	12	25	38	13	88	0	25	25	0	25	13	13	0	25	50	25
Reliability	Website Accessibility	100	100	100	100	100	100	100	100	100	100	100	100	100	100	100	100	100
	Survey Transparency Rate	0	38	0	0	100	100	0	75	100	100	100	100	0	100	100	100	0
	Message Response Transparency Rate	26	3	2	2	3	5	90	0	99	50	3	100	100	100	53	47	2
	Number of Government Service items	100	73	45	26	48	39	3	27	22	34	39	52	15	38	1	0	15
	Average Transaction per User Rate	5	8	0	0	11	0	0	0	100	0	78	1	0	0	0	0	1
	Total Information Releases	7	100	21	9	5	3	94	3	1	0	1	4	2	0	2	7	4
Responsiveness	Page Design	0	100	100	0	100	0	100	100	100	0	0	0	0	0	0	0	0
	Average Response Time for Public Messages	0	0	100	100	0	100	0	0	100	0	100	100	100	0	0	0	0
	Public Message Processing Rate	100	100	100	86	0	100	100	100	92	100	100	100	100	100	100	100	100
	Public Response Rate in Online Interviews	71	100	0	0	57	100	0	0	0	0	100	0	0	100	0	100	100

Dimension	Sub-dimension	Chongqing	Shanghai	Beijing	Chengdu	Guangzhou	Shenzhen	Tianjin	Wuhan	Xian	Suzhou	Zhengzhou	Hangzhou	Linyi	Dongguan	Shijiazhuang	Changsha	Qingdao
Assurance	Security Detection and Early Warning Mechanism	100	100	100	100	100	100	100	100	100	100	100	100	100	100	100	100	100
	Website Administrator	100	100	100	100	100	100	100	100	100	100	100	100	100	100	100	100	100
	Frequency of Security Inspections and Assessments	11	16	33	47	47	38	20	11	11	11	11	2	7	2	0	100	47
	Emergency Response Drills	100	100	100	100	100	100	100	100	100	100	100	100	100	100	100	100	100
	Cross-Browser and Cross-Device Compatibility	100	100	100	100	100	100	100	100	0	0	100	100	100	100	100	0	0
Empathy	Online Service Completion Rate	35	24	55	0	100	73	100	90	2	100	100	100	52	100	100	28	75
	Website Special Features	40	80	60	0	60	40	0	0	20	20	20	60	40	20	20	0	0
	Weibo Information Release Volume	5	27	16	100	28	41	50	12	12	22	0	46	9	2	24	0	28
	WeChat Information Release Volume	37	25	14	36	26	22	68	0	27	16	31	28	21	34	21	5	100
	Cross-Platform Interoperability	0	0	0	13	100	0	0	67	7	0	0	33	0	0	0	0	0
	Service Item Catalog	100	100	100	100	100	100	100	100	100	100	100	100	100	100	100	100	100
	Intelligent Q&A	0	0	0	0	100	100	0	0	0	0	0	0	0	100	0	100	0
	Number of Public Opinions Received	2	100	20	0	3	3	1	0	0	78	2	3	0	0	1	25	0
	Annual Volume of Policy Interpretations Published	4	7	2	0	28	9	9	3	1	72	1	11	6	100	7	0	39
	Number of Public Concerns or Major Issues Addressed	1	26	2	100	7	6	0	8	13	2	0	5	1	29	3	1	2

Supplementary Table 5 Value of each city in 2021

Dimension	Sub-dimension	Chongqing	Shanghai	Beijing	Chengdu	Guangzhou	Shenzhen	Tianjin	Wuhan	Xian	Suzhou	Zhengzhou	Hangzhou	Linyi	Dongguan	Shijiazhuang	Changsha	Qingdao
Tangibility	Unified Platform for Interaction	100	100	100	100	100	100	100	100	100	100	100	100	100	100	0	100	100
	Number of Public Surveys Conducted	70	70	20	10	40	100	15	20	10	10	10	65	0	0	15	15	0
	Number of Online Expert Interviews	14	100	0	0	14	100	0	14	0	0	14	14	0	5	0	0	29
	Volume of Public Messages Received	14	52	24	9	21	16	9	0	100	5	9	62	3	1	10	7	21
	Number of Specialized Sections Maintained	42	22	22	19	11	53	22	8	28	0	14	22	19	100	19	17	14
	Number of Newly Established Specialized Sections	13	0	75	25	25	100	50	0	25	13	0	25	63	13	38	25	50
Reliability	Website Accessibility	100	100	100	100	100	100	100	100	100	100	100	100	100	100	100	100	100
	Survey Transparency Rate	7	79	75	0	100	100	0	0	100	100	0	92	0	0	100	100	0
	Message Response Transparency Rate	6	9	2	5	2	4	59	0	89	44	2	35	100	100	6	9	0
	Number of Government Service items	75	33	33	0	38	33	5	22	38	25	30	36	14	28	0	100	11
	Average Transaction per User Rate	1	2	1	0	3	100	0	0	37	0	12	0	0	0	0	1	0
	Total Information Releases	7	100	12	7	2	2	3	3	1	0	0	4	2	0	1	5	7
Responsiveness	Page Design	100	100	100	100	100	0	100	100	100	0	0	0	0	0	0	0	0
	Average Response Time for Public Messages	0	0	100	0	0	100	0	0	100	0	100	100	100	0	100	0	0
	Public Message Processing Rate	100	100	100	100	100	100	100	0	89	100	100	100	100	100	100	100	100
	Public Response Rate in Online Interviews	100	100	0	0	0	100	0	0	0	0	100	0	0	100	0	0	100

Dimension	Sub-dimension	Chongqing	Shanghai	Beijing	Chengdu	Guangzhou	Shenzhen	Tianjin	Wuhan	Xian	Suzhou	Zhengzhou	Hangzhou	Linyi	Dongguan	Shijiazhuang	Changsha	Qingdao
Assurance	Security Detection and Early Warning Mechanism	100	100	100	100	100	100	100	100	100	100	100	100	100	100	100	100	100
	Website Administrator	100	100	100	100	100	100	100	100	100	100	100	100	100	100	100	100	100
	Frequency of Security Inspections and Assessments	1	1	3	3	3	2	2	100	1	1	1	0	0	0	0	5	3
	Emergency Response Drills	100	100	100	100	100	100	100	100	100	0	100	100	100	100	100	100	100
	Cross-Browser and Cross-Device Compatibility	100	100	100	100	100	100	100	100	0	0	100	100	100	100	100	0	0
Empathy	Online Service Completion Rate	18	77	100	0	97	99	100	100	67	100	100	100	10	100	11	3	97
	Website Special Features	60	80	60	0	60	40	0	0	20	0	80	60	40	20	20	60	20
	Weibo Information Release Volume	5	23	64	100	30	43	44	5	13	23	0	4	4	0	23	0	23
	WeChat Information Release Volume	64	38	48	42	41	41	100	0	43	24	70	31	32	31	54	6	88
	Cross-Platform Interoperability	0	0	0	19	88	0	4	12	6	0	0	100	74	0	0	0	22
	Service Item Catalog	100	100	100	100	100	100	100	100	100	100	100	100	100	100	100	100	100
	Intelligent Q&A	0	0	0	0	100	100	0	0	0	0	0	100	0	0	0	100	0
	Number of Public Opinions Received	2	3	33	0	4	5	9	0	3	39	0	3	0	0	1	100	0
	Annual Volume of Policy Interpretations Published	70	100	21	0	32	15	9	8	9	28	6	19	21	4	25	6	13
	Number of Public Concerns or Major Issues Addressed	0	97	5	5	7	16	0	9	9	0	7	20	2	100	9	3	6

Supplementary Table 6 Value of each city in 2022

Dimension	Sub-dimension	Chongqing	Shanghai	Beijing	Chengdu	Guangzhou	Shenzhen	Tianjin	Wuhan	Xian	Suzhou	Zhengzhou	Hangzhou	Linyi	Dongguan	Shijiazhuang	Changsha	Qingdao
Tangibility	Unified Platform for Interaction	100	100	100	100	100	100	100	100	100	100	100	100	100	100	100	100	100
	Number of Public Surveys Conducted	100	37	47	11	26	79	16	5	16	26	5	37	21	0	16	16	0
	Number of Online Expert Interviews	2	34	0	0	5	100	0	12	0	0	5	3	0	2	0	0	12
	Volume of Public Messages Received	25	65	42	0	35	44	27	7	100	8	5	73	4	2	11	5	38
	Number of Specialized Sections Maintained	100	72	78	67	33	89	0	33	44	11	33	56	61	61	50	33	56
	Number of Newly Established Specialized Sections	9	18	9	18	9	73	100	9	0	0	0	0	27	0	0	0	27
Reliability	Website Accessibility	100	100	100	100	100	100	100	100	100	100	100	100	100	100	100	100	100
	Survey Transparency Rate	0	100	100	0	100	40	0	0	67	100	100	100	100	0	100	100	0
	Message Response Transparency Rate	3	100	1	100	1	1	37	0	78	20	2	89	82	100	1	100	0
	Number of Government Service items	100	45	49	14	43	48	6	26	45	33	45	48	14	42	0	25	13
	Average Transaction per User Rate	1	3	0	1	3	100	0	0	0	0	5	0	0	0	35	0	0
	Total Information Releases	6	100	10	7	4	3	6	1	1	0	0	5	1	1	0	1	6
	Page Design	100	100	100	100	100	100	100	100	100	0	0	0	0	0	0	100	0
Responsiveness	Average Response Time for Public Messages	0	0	0	0	0	100	0	0	100	0	0	100	100	0	100	100	0
	Public Message Processing Rate	100	100	100	100	100	100	0	100	34	97	100	100	100	100	100	100	100
	Public Response Rate in Online Interviews	0	100	0	0	100	100	0	0	0	0	100	0	0	100	0	0	100

Dimension	Sub-dimension	Chongqing	Shanghai	Beijing	Chengdu	Guangzhou	Shenzhen	Tianjin	Wuhan	Xian	Suzhou	Zhengzhou	Hangzhou	Linyi	Dongguan	Shijiazhuang	Changsha	Qingdao
	Security Detection and Early Warning Mechanism	100	100	100	100	100	100	100	100	100	100	100	100	100	100	100	100	100
	Website Administrator	100	100	100	100	100	100	100	100	100	100	100	100	100	100	100	100	100
Assurance	Frequency of Security Inspections and Assessments	58	26	58	16	58	53	63	58	11	16	16	5	5	0	16	100	58
	Emergency Response Drills	100	100	100	100	100	100	100	100	100	0	100	100	100	100	100	100	100
	Cross-Browser and Cross-Device Compatibility	100	100	100	100	100	100	100	100	0	0	100	100	100	100	100	0	0
	Online Service Completion Rate	35	82	100	93	100	100	100	100	96	0	100	100	37	100	100	100	95
	Website Special Features	60	80	60	20	60	40	20	40	20	0	80	40	40	20	20	40	20
	Weibo Information Release Volume	2	21	29	100	52	45	54	5	6	5	0	14	12	0	27	0	19
	WeChat Information Release Volume	40	28	44	48	41	38	100	0	29	31	57	28	54	35	47	4	69
	Cross-Platform Interoperability	0	0	0	13	100	0	0	13	6	0	0	87	95	0	0	0	19
Empathy	Service Item Catalog	100	100	100	100	100	100	100	100	100	100	100	100	100	100	100	100	100
	Intelligent Q&A	0	100	100	0	100	100	0	0	100	0	0	100	0	0	0	100	0
	Number of Public Opinions Received	0	1	0	0	12	1	0	0	2	0	0	4	0	0	1	100	0
	Annual Volume of Policy Interpretations Published	20	1000	5	1	22	9	16	0	2	13	1	10	12	1	8	0	4
	Number of Public Concerns or Major Issues Addressed	0	80	31	2	1	6	0	3	0	1	1	28	0	100	1	1	2

Supplementary Table 7 Value of each city in 2023

Dimension	Sub-dimension	Chongqing	Shanghai	Beijing	Chengdu	Guangzhou	Shenzhen	Tianjin	Wuhan	Xian	Suzhou	Zhengzhou	Hangzhou	Linyi	Dongguan	Shijiazhuang	Changsha	Qingdao
Tangibility	Unified Platform for Interaction	100	100	100	100	100	100	100	100	100	100	100	100	100	100	100	100	100
	Number of Public Surveys Conducted	95	21	100	21	26	63	0	16	21	21	5	42	11	0	5	16	5
	Number of Online Expert Interviews	7	100	0	0	20	27	0	13	0	0	27	13	0	7	0	0	27
	Volume of Public Messages Received	13	14	17	3	8	18	4	1	100	2	2	16	2	0	2	1	12
	Number of Specialized Sections Maintained	100	53	71	59	41	65	24	18	24	0	29	53	24	53	47	24	41
	Number of Newly Established Specialized Sections	14	29	29	29	14	100	29	14	0	0	14	14	0	0	14	14	43
Reliability	Website Accessibility	100	100	100	100	100	100	100	100	100	100	100	100	100	100	100	100	100
	Survey Transparency Rate	11	100	95	0	100	100	0	0	100	75	100	100	100	0	0	100	100
	Message Response Transparency Rate	3	36	1	3	1	2	60	1	90	25	5	98	33	100	12	100	0
	Number of Government Service items	100	41	62	30	81	93	5	65	72	62	87	82	22	93	0	49	15
	Average Transaction per User Rate	23	51	44	19	100	9	0	11	0	0	16	0	0	0	0	6	0
	Total Information Releases	7	100	21	17	10	3	4	0	4	1	0	7	2	1	2	2	2
Responsiveness	Page Design	100	100	100	100	100	100	100	100	100	0	0	100	0	0	0	100	100
	Average Response Time for Public Messages	0	0	0	0	100	100	0	0	0	0	0	100	100	0	100	100	0
	Public Message Processing Rate	100	100	91	71	100	100	100	100	0	100	100	100	100	100	100	100	100
	Public Response Rate in Online Interviews	0	100	0	0	100	100	100	0	0	0	100	0	0	100	0	0	100

Dimension	Sub-dimension	Chongqing	Shanghai	Beijing	Chengdu	Guangzhou	Shenzhen	Tianjin	Wuhan	Xian	Suzhou	Zhengzhou	Hangzhou	Linyi	Dongguan	Shijiazhuang	Changsha	Qingdao
Assurance	Security Detection and Early Warning Mechanism	100	100	100	100	100	100	100	100	100	100	100	100	100	100	100	100	100
	Website Administrator	100	100	100	100	100	100	100	100	100	100	100	100	100	100	100	100	100
	Frequency of Security Inspections and Assessments	50	42	92	25	92	67	100	92	67	25	25	0	8	0	25	92	92
	Emergency Response Drills	100	100	100	100	100	100	100	100	100	100	100	100	100	100	100	100	100
	Cross-Browser and Cross-Device Compatibility	100	100	100	100	100	100	100	100	0	0	100	100	100	100	100	0	0
	Online Service Completion Rate	100	92	100	87	100	100	100	77	94	0	100	100	57	100	88	96	97
Empathy	Website Special Features	60	80	60	20	60	40	40	40	20	20	60	60	40	40	20	40	20
	Weibo Information Release Volume	3	15	24	100	19	42	54	5	5	66	0	91	9	0	33	0	17
	WeChat Information Release Volume	42	13	37	33	32	30	87	0	33	34	41	34	57	23	59	2	100
	Cross-Platform Interoperability	0	0	0	18	89	0	0	0	4	0	0	51	100	0	0	0	30
	Service Item Catalog	100	100	100	100	100	100	100	100	100	100	100	100	100	100	100	100	100
	Intelligent Q&A	0	100	100	0	100	100	0	0	100	0	0	100	0	100	0	100	100
	Number of Public Opinions Received	15	47	18	0	34	74	0	0	10	0	0	33	0	0	1	100	6
	Annual Volume of Policy Interpretations Published	21	100	92	6	38	21	13	3	25	25	6	17	19	0	11	11	16
	Number of Public Concerns or Major Issues Addressed	0	19	58	46	19	46	0	42	0	0	8	100	0	0	0	12	8

Supplementary Table 8 Value of each city in 2024

Dimension	Sub-dimension	Chongqing	Shanghai	Beijing	Chengdu	Guangzhou	Shenzhen	Tianjin	Wuhan	Xian	Suzhou	Zhengzhou	Hangzhou	Linyi	Dongguan	Shijiazhuang	Changsha	Qingdao
Tangibility	Unified Platform for Interaction	100	100	100	100	100	100	100	100	100	100	100	100	100	100	100	100	100
	Number of Public Surveys Conducted	100	44	67	33	44	78	44	61	22	22	11	67	0	0	6	6	11
	Number of Online Expert Interviews	5	100	5	0	20	30	95	5	0	0	15	15	0	5	0	0	10
	Volume of Public Messages Received	15	17	23	3	6	45	4	2	100	2	3	21	1	0	2	1	25
	Number of Specialized Sections Maintained	100	53	59	65	59	65	65	41	35	0	29	29	24	53	12	24	59
	Number of Newly Established Specialized Sections	13	25	25	38	38	100	75	0	0	0	0	13	0	0	0	0	38
Reliability	Website Accessibility	100	100	100	100	100	100	100	100	100	100	100	100	100	100	100	100	100
	Survey Transparency Rate	100	100	100	0	100	100	100	36	100	75	100	100	0	0	0	100	100
	Message Response Transparency Rate	3	4	1	3	2	1	49	1	96	21	2	83	68	100	13	100	0
	Number of Government Service items	95	49	64	27	95	100	6	67	74	64	88	83	25	78	0	64	13
	Average Transaction per User Rate	25	100	30	20	94	7	0	93	0	0	15	0	0	0	0	31	0
	Total Information Releases	5	100	17	15	8	2	5	0	3	1	0	5	1	1	2	3	1
Responsiveness	Page Design	100	100	100	100	100	100	100	100	100	0	0	100	0	0	0	100	100
	Average Response Time for Public Messages	0	0	0	0	100	0	0	0	0	0	0	0	100	0	100	100	0
	Public Message Processing Rate	100	100	75	70	100	100	0	100	65	100	100	100	100	100	100	100	100
	Public Response Rate in Online Interviews	0	97	0	0	100	100	0	0	0	0	100	0	0	100	0	0	100

Dimension	Sub-dimension	Chongqing	Shanghai	Beijing	Chengdu	Guangzhou	Shenzhen	Tianjin	Wuhan	Xian	Suzhou	Zhengzhou	Hangzhou	Linyi	Dongguan	Shijiazhuang	Changsha	Qingdao
Assurance	Security Detection and Early Warning Mechanism	100	100	100	100	100	100	100	100	100	100	100	100	100	100	100	100	100
	Website Administrator	100	100	100	100	100	100	100	100	100	100	100	100	100	100	100	100	100
	Frequency of Security Inspections and Assessments	69	13	69	0	6	50	75	69	100	19	19	0	6	0	19	69	69
	Emergency Response Drills	100	100	100	0	100	100	100	100	100	100	100	100	100	100	100	100	100
	Cross-Browser and Cross-Device Compatibility	100	100	100	100	100	100	100	100	0	0	100	100	100	100	100	0	0
	Online Service Completion Rate	100	69	100	100	89	100	100	77	93	0	100	100	57	100	73	100	95
Empathy	Website Special Features	60	60	60	40	60	40	40	40	20	20	60	60	40	40	20	40	20
	Weibo Information Release Volume	2	17	94	100	18	41	31	4	3	51	0	51	2	0	24	0	15
	WeChat Information Release Volume	31	17	33	23	35	30	63	0	33	39	16	39	39	20	55	8	100
	Cross-Platform Interoperability	0	0	0	35	100	0	5	0	9	0	0	61	13	0	0	0	72
	Service Item Catalog	100	100	100	100	100	100	100	100	100	100	100	100	100	100	100	100	100
	Intelligent Q&A	0	100	100	0	100	100	0	0	100	0	0	100	0	100	0	100	100
	Number of Public Opinions Received	0	19	1	0	5	2	0	0	100	0	1	0	0	0	0	1	1
	Annual Volume of Policy Interpretations Published	8	13	100	2	21	9	0	0	3	6	1	8	2	1	2	2	6
	Number of Public Concerns or Major Issues Addressed	0	15	100	15	45	75	0	0	0	0	40	35	0	0	0	15	5

2 Calculation of Example

The following is an example of the ranking calculation process for each city in the tangibility dimension in 2024：

（1）Construction of a standardized matrix (See Formula 1 in the manuscript)

Dimension	Sub-dimension	Chongqing	Shanghai	Beijing	Chengdu	Guangzhou	Shenzhen	Tianjin	Wuhan	Xian	Suzhou	Zhengzhou	Hangzhou	Linyi	Dongguan	Shijiazhuang	Changsha	Qingdao
Tangibility	Unified Platform for Interaction	100	100	100	100	100	100	100	100	100	100	100	100	100	100	100	100	100
	Number of Public Surveys Conducted	100	44	67	33	44	78	44	61	22	22	11	67	0	0	6	6	11
	Number of Online Expert Interviews	5	100	5	0	20	30	95	5	0	0	15	15	0	5	0	0	10
	Volume of Public Messages Received	15	17	23	3	6	45	4	2	100	2	3	21	1	0	2	1	25
	Number of Specialized Sections Maintained	100	53	59	65	59	65	65	41	35	0	29	29	24	53	12	24	59
	Number of Newly Established Specialized Sections	13	25	25	38	38	100	75	0	0	0	0	13	0	0	0	0	38

（2）Data standardization (See Formula 2 in the manuscript)

Dimension	Sub-dimension	Chongqing	Shanghai	Beijing	Chengdu	Guangzhou	Shenzhen	Tianjin	Wuhan	Xian	Suzhou	Zhengzhou	Hangzhou	Linyi	Dongguan	Shijiazhuang	Changsha	Qingdao
Tangibility	Volume of Public Messages Received	0.1501	0.1701	0.2301	0.0301	0.0601	0.4501	0.0401	0.0201	1.0001	0.0201	0.0301	0.2101	0.0101	0.0001	0.0201	0.0101	0.2501
	Number of Public Surveys Conducted	1.0001	0.4401	0.6701	0.3301	0.4401	0.7801	0.4401	0.6101	0.2201	0.2201	0.1101	0.6701	0.0001	0.0001	0.0601	0.0601	0.1101
	Number of Online Expert Interviews	0.0501	1.0001	0.0501	0.0001	0.2001	0.3001	0.9501	0.0501	0.0001	0.0001	0.1501	0.1501	0.0001	0.0501	0.0001	0.0001	0.1001
	Number of Specialized Sections Maintained	1.0001	0.5301	0.5901	0.6501	0.5901	0.6501	0.6501	0.4101	0.3501	0.0001	0.2901	0.2901	0.2401	0.5301	0.1201	0.2401	0.5901

Dimension	Sub-dimension	Chongqing	Shanghai	Beijing	Chengdu	Guangzhou	Shenzhen	Tianjin	Wuhan	Xian	Suzhou	Zhengzhou	Hangzhou	Linyi	Dongguan	Shijiazhuang	Changsha	Qingdao
	Number of Newly Established Specialized Sections	0.1301	0.2501	0.2501	0.3801	0.3801	1.0001	0.7501	0.0001	0.0001	0.0001	0.0001	0.1301	0.0001	0.0001	0.0001	0.0001	0.3801

（3）Calculation of the proportion of the standardized values of each city in each indicator(See Formula 3 in the manuscript)

Dimension	Sub-dimension	Chongqing	Shanghai	Beijing	Chengdu	Guangzhou	Shenzhen	Tianjin	Wuhan	Xian	Suzhou	Zhengzhou	Hangzhou	Linyi	Dongguan	Shijiazhuang	Changsha	Qingdao
Tangibility	Volume of Public Messages Received	0.055558	0.06296	0.085169	0.011141	0.022245	0.166599	0.014843	0.00744	0.370174	0.00744	0.011141	0.077766	0.003738	3.70E-05	0.00744	0.003738	0.092571
	Number of Public Surveys Conducted	0.162309	0.071425	0.108752	0.053573	0.071425	0.126605	0.071425	0.099015	0.035721	0.035721	0.017868	0.108752	1.60E-05	1.60E-05	0.009754	0.009754	0.017868
	Number of Online Expert Interviews	0.016417	0.327719	0.016417	3.30E-05	0.06557	0.098339	0.311335	0.016417	3.30E-05	3.30E-05	0.049186	0.049186	3.30E-05	0.016417	3.30E-05	3.30E-05	0.032801
	Number of Specialized Sections Maintained	0.129518	0.068651	0.076421	0.084191	0.076421	0.084191	0.084191	0.05311	0.04534	1.30E-05	0.037569	0.037569	0.031094	0.068651	0.015554	0.031094	0.076421
	Number of Newly Established Specialized Sections	0.035627	0.068489	0.068489	0.104089	0.104089	0.273872	0.205411	2.70E-05	2.70E-05	2.70E-05	2.70E-05	0.035627	2.70E-05	2.70E-05	2.70E-05	2.70E-05	0.104089

（4）Calculation of the index information entropy value(See Formula 4 in the manuscript)

Dimension	Sub-dimension	Entropy value
Tangibility	Volume of Public Messages Received	0.716052
	Number of Public Surveys Conducted	0.869387
	Number of Online Expert Interviews	0.640941
	Number of Specialized Sections Maintained	0.942597
	Number of Newly Established Specialized Sections	0.703606

（5）Calculation of the index information utility value(See Formula 5 in the manuscript)

Dimension	Sub-dimension	Information utility value
	Volume of Public Messages Received	0.283948
	Number of Public Surveys Conducted	0.130613
Tangibility	Number of Online Expert Interviews	0.359059
	Number of Specialized Sections Maintained	0.057403
	Number of Newly Established Specialized Sections	0.296394

（6）Calculation of the weight of evaluation indicators(See Formula 6 in the manuscript)

Dimension	Sub-dimension	Weight
	Volume of Public Messages Received	0.046179
	Number of Public Surveys Conducted	0.021242
Tangibility	Number of Online Expert Interviews	0.058395
	Number of Specialized Sections Maintained	0.009336
	Number of Newly Established Specialized Sections	0.048203

（7）Calculation of the weighted decision matrix(See Formula 7 in the manuscript)

Dimension	Sub-dimension	Chongqing	Shanghai	Beijing	Chengdu	Guangzhou	Shenzhen	Tianjin	Wuhan	Xian	Suzhou	Zhengzhou	Hangzhou	Linyi	Dongguan	Shijiazhuang	Changsha	Qingdao
Tangibility	Volume of Public Messages Received	0.006932	0.007855	0.010626	0.00139	0.002775	0.020785	0.001852	0.000928	0.046184	0.000928	0.00139	0.009702	0.000466	5.00E-06	0.000928	0.000466	0.011549
	Number of Public Surveys Conducted	0.021244	0.009349	0.014234	0.007012	0.009349	0.016571	0.009349	0.01296	0.004675	0.004675	0.002339	0.014234	2.00E-06	2.00E-06	0.001277	0.001277	0.002339
	Number of Online Expert Interviews	0.002926	0.058401	0.002926	6.00E-06	0.011685	0.017524	0.055481	0.002926	6.00E-06	6.00E-06	0.008765	0.008765	6.00E-06	0.002926	6.00E-06	6.00E-06	0.005845
	Number of Specialized Sections Maintained	0.009337	0.004949	0.005509	0.006069	0.005509	0.006069	0.006069	0.003829	0.003268	1.00E-06	0.002708	0.002708	0.002241	0.004949	0.001121	0.002241	0.005509
	Number of Newly Established Specialized Sections	0.006271	0.012056	0.012056	0.018322	0.018322	0.048208	0.036157	5.00E-06	5.00E-06	5.00E-06	5.00E-06	0.006271	5.00E-06	5.00E-06	5.00E-06	5.00E-06	0.018322

（8）Calculation of the positive and negative ideal solutions of each city in each dimension((See Formulas 8 and 9 in the manuscript))

Dimension	Sub-dimension	positive ideal solution	negative ideal solution
Tangibility	Volume of Public Messages Received	0.046184	5.00E-06
	Number of Public Surveys Conducted	0.021244	2.00E-06
	Number of Online Expert Interviews	0.058401	6.00E-06
	Number of Specialized Sections Maintained	0.009337	1.00E-06
	Number of Newly Established Specialized Sections	0.048208	5.00E-06

（9）Calculation of the positive and negative ideal distances and ideal closeness coefficient of each city in each dimension, and rank them according to the ideal closeness coefficient (See Formulas 10 to 12 in the manuscript.)

City	negative ideal distances	negative ideal distances	ideal closeness coefficient	Rank
Chongqing	0.079856	0.025182	0.239745	7
Shanghai	0.054193	0.061063	0.529803	3
Beijing	0.075582	0.022348	0.228206	8
Chengdu	0.080764	0.020577	0.203045	10
Guangzhou	0.071526	0.02444	0.25467	5
Shenzhen	0.048461	0.058083	0.545155	2
Tianjin	0.047658	0.067172	0.584971	1
Wuhan	0.08688	0.013854	0.137529	11
Xian	0.077749	0.04653	0.3744	4
Suzhou	0.09024	0.004764	0.050141	14
Zhengzhou	0.084824	0.009562	0.101308	12
Hangzhou	0.075143	0.020492	0.214274	9
Linyi	0.091243	0.002288	0.024459	16
Dongguan	0.089465	0.005745	0.060341	13

City	negative ideal distances	negative ideal distances	ideal closeness coefficient	Rank
Shijiazhuang	0.090817	0.001932	0.02083	17
Changsha	0.090954	0.002619	0.027986	15
Qingdao	0.072297	0.02321	0.243019	6
